# *HSP101*‐encoding NEO‐TETRAPLOID RICE FERTILITY GENE 1 regulates tapetum development through interaction with SAPK2 in polyploid rice

**DOI:** 10.1111/jipb.70218

**Published:** 2026-03-11

**Authors:** Lichong Cao, Weicong Huang, Hang Yu, Sanglin Liu, Jianmin Yin, Zijun Lu, Jinwen Wu, Xiangdong Liu

**Affiliations:** ^1^ State Key Laboratory for Conservation and Utilization of Subtropical Agro‐Bioresources, Guangdong Laboratory for Lingnan Modern Agriculture South China Agricultural University Guangzhou 510642 China; ^2^ Guangdong Provincial Key Laboratory of Plant Molecular Breeding, Guangdong Base Bank for Lingnan Rice Germplasm Resources, College of Agriculture South China Agricultural University Guangzhou 510642 China; ^3^ Key Laboratory for Enhancing Resource Use Efficiency of Crops in South China Ministry of Agriculture and Rural Affairs Guangzhou 510642 China; ^4^ Rice Research Institute Guangdong Academy of Agricultural Sciences Guangzhou 510640 China

**Keywords:** abscisic acid, neo‐tetraploid rice, *NTRF1*, pollen development, programmed cell death, reactive oxygen species, rice (*Oryza sativa* L.)

## Abstract

A novel allelic variant of the heat shock protein 101, designated neo‐tetraploid rice fertility gene 1 (*NTRF1*), has been identified and is implicated in regulating fertility in neo‐tetraploid rice (NTR); however, its regulatory mechanism remains unclear. In this study, we identified the *ntrf1* mutant and demonstrated that its significantly reduced seed‐setting rate was due to pollen developmental defects. Mechanistically, *NTRF1* deficiency disrupts reactive oxygen species (ROS) homeostasis in anthers, thereby delaying the progression of programmed cell death (PCD) in tapetal cells. RNA‐seq analysis of mutant anthers revealed dysregulated expression of abscisic acid (ABA) signaling components (*OsPP2C49*, *OsbZIP23*) and ROS‐related genes (*OsRBOH1*, *OsRBOH8*), along with a significant downregulation of key tapetal developmental regulators (*OsGAmyb*, *CYP703A3*). Integrated multi‐omics analysis showed that the reduced pollen viability in the *ntrf1* mutant is associated with the pyruvate metabolic pathway. Protein interaction assays confirmed that NTRF1 directly binds SAPK2, a core kinase in ABA signaling transduction. This interaction explained how exogenous ABA application partially restored the reduced seed‐setting rate in *ntrf1* mutants. Collectively, our findings elucidated an *NTRF1*‐centered regulatory network that coordinates ABA signaling with ROS homeostasis to ensure timely tapetal PCD and subsequent pollen maturation. This study provides valuable molecular targets for advancing the genetic improvement of polyploid rice.

## Graphical abstract

In neo‐tetraploid rice, the NTRF1–SAPK2 heat shock protein–kinase module coordinates abscisic acid signaling with reactive oxygen species homeostasis to ensure timely tapetal programmed cell death and subsequent pollen maturation, providing a resource for the genetic improvement of polyploid rice.

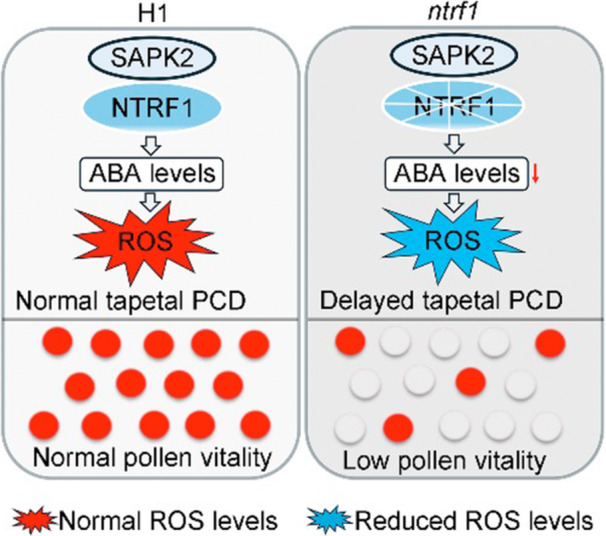

## INTRODUCTION

Rice (*Oryza sativa* L.) is one of the world's most crucial cereal crops, with over half of the global population depending on it as their staple food (Sasaki et al., 2002; Wang et al., 2020). The growth and stability of rice production are critically important for ensuring food security. Advancements in breeding techniques are a vital strategy for attaining sustainable increases in rice production (Yu et al., 2021). Polyploidization is a crucial evolutionary mechanism that drives the diversification of angiosperms (Soltis et al., 2015; Edger et al., 2018; Wu et al., 2021). Polyploid plants offer numerous advantages, including robust growth, enhanced stress tolerance, larger fruit size, improved nutritional content, and strong heterosis, making them a promising breeding strategy (Comai, 2005; Chao et al., 2013; Corneillie et al., 2019; Liu et al., 2023). Many critical crops, such as wheat (*Triticum aestivum*) and sugarcane (*Saccharum officinarum*), are polyploids, and polyploidization has proven effective in enhancing other crops as well (Feldman et al., 2012; Liu et al., 2023; Zhang et al., 2025).

Autotetraploid rice (ATR) is a useful germplasm that, like other polyploid plants, possesses several advantageous traits, including larger grain size, higher grain weight and protein content (Chen et al., 2021; Lv et al., 2025). However, low fertility presents a significant challenge in ATR research (Bao and Yan, 1956; Wu et al., 2015; Li et al., 2017). Several factors have been identified as influencing ATR fertility, including abnormal chromosome behaviors during meiosis, aberrant gene expression, and epigenetic changes (Lu et al., 2020; Wu et al., 2020; Liu et al., 2023). Since the 21st century, China has made substantial progress in deciphering the genetic basis of low fertility in ATR, successfully developing high‐fertility tetraploid varieties, such as polyploid meiosis stability rice (PMeS) and neo‐tetraploid rice (NTR) (Guo et al., 2017; Chen et al., 2021; Liu et al., 2023). Two NTR lines, Huaduo 1 and Huaduo 2, were granted national plant variety rights in 2016 (Liu et al., 2023). These breakthroughs have effectively overcome the long‐standing fertility challenges in ATR, opening up promising avenues for agricultural applications.

To clarify the genetic mechanisms underlying high fertility in NTR, our previous research conducted comparative genomic analysis using re‐sequencing data from NTR, ATR lines, and diploid rice 3 K re‐sequencing data. This work successfully identified a novel allelic variant of *HSP101*, named neo‐tetraploid rice fertility gene 1 (*NTRF1*), as a key regulator of fertility in NTR (Yu et al., 2021). *NTRF1* harbors a specific SNP variation, namely a C‐to‐G single‐nucleotide polymorphism (SNP) at the seventh nucleotide position in its first exon, and exhibits specific expression in anther tissues during meiosis under normal conditions (Yu et al., 2021). However, the precise regulatory mechanisms are still unclear.

Heat shock proteins (HSPs), central components of the stress response, are categorized into several subfamilies, including HSP40, HSP60, HSP70, HSP90, HSP100, and small HSPs (Ren et al., 2024). As a member of the HSP100/ClpB family, *HSP101* is a key response factor for heat tolerance (Hong and Vierling, 2001; Lin et al., 2014; Kim et al., 2023). Emerging evidence indicates that *HSP101* plays additional roles in plant development. In *Arabidopsis*, *HSP101* regulates flowering time, with knockout lines exhibiting delayed flowering and overexpression lines showing early flowering (Qin et al., 2021). In rice, *FLO24* (encoding HSP101) influences starch biosynthesis and endosperm development (Wu et al., 2024). *HSP101* can also act as a regulatory factor to safeguard male meiosis against the impacts of heat stress (Li et al., 2025). Notably, maize *ZmHSP101* is involved in meiotic DNA double‐strand break (DSB) repair (Li et al., 2022b), suggesting conserved reproductive functions of *HSP101* across plants. However, the molecular mechanisms by which it regulates fertility in tetraploid rice remain to be clarified.

Recent studies have revealed connections between HSPs and ROS (Ul Haq et al., 2019). Under heat stress, HSPs assist in scavenging excess ROS by upregulating the antioxidant enzyme system, while also utilizing ROS as signaling molecules to activate their own expression (Qu et al., 2013). Similarly, there is a strong connection between ROS generation and the ABA signaling pathway (Li et al., 2022a; Zhao et al., 2023). In rice, *OsDMI3*‐mediated phosphorylation of *OsRBOHb* activates NADPH oxidase activity in a sucrose non‐fermenting‐related kinase *SAPK8*/*9*/*10*‐dependent manner (Wang et al., 2023). Additionally, ABA perception by RCAR receptors activates SnRK2 kinases, which stimulate apoplastic ROS generation to regulate stomatal closure (Liu et al., 2022a). Furthermore, during rice anther development, *SAPK2*‐mediated ROS homeostasis is crucial for normal PCD (Zhao et al., 2023), a key component of pollen development (Chen et al., 2023b; Zhou et al., 2024; Shi et al., 2024a). Although both HSPs and ABA signaling independently regulate ROS (Zhou et al., 2022; Xiang et al., 2024), the potential crosstalk between them in tapetal PCD remains unexplored.

In our previous studies, it was preliminarily determined that *NTRF1* plays a critical role in regulating fertility in NTR (Yu et al., 2021). In this study, we systematically investigated the molecular mechanisms by which *NTRF1* regulates fertility in NTR. Under normal conditions, *ntrf1* mutants showed a significantly lower seed‐setting rate in NTR, while the loss of *HSP101* function did not affect the seed‐setting rate in diploid rice. Cytological analysis showed that the *ntrf1* mutant significantly reduced pollen viability and delayed the progression of PCD in the tapetum cells. Mechanistic analyses revealed the physical interaction between NTRF1 and SAPK2, and exogenous ABA application partially restored the ROS levels and pollen viability that resulted from the loss of *NTRF1* function. These findings not only deepen our understanding of the regulatory mechanisms underlying rice fertility but also provide a scientific basis for breeding strategies in polyploid rice.

## RESULTS

### The *ntrf1* mutant shows a low seed‐setting rate in neo‐tetraploid rice (NTR)

**Figure 1:**
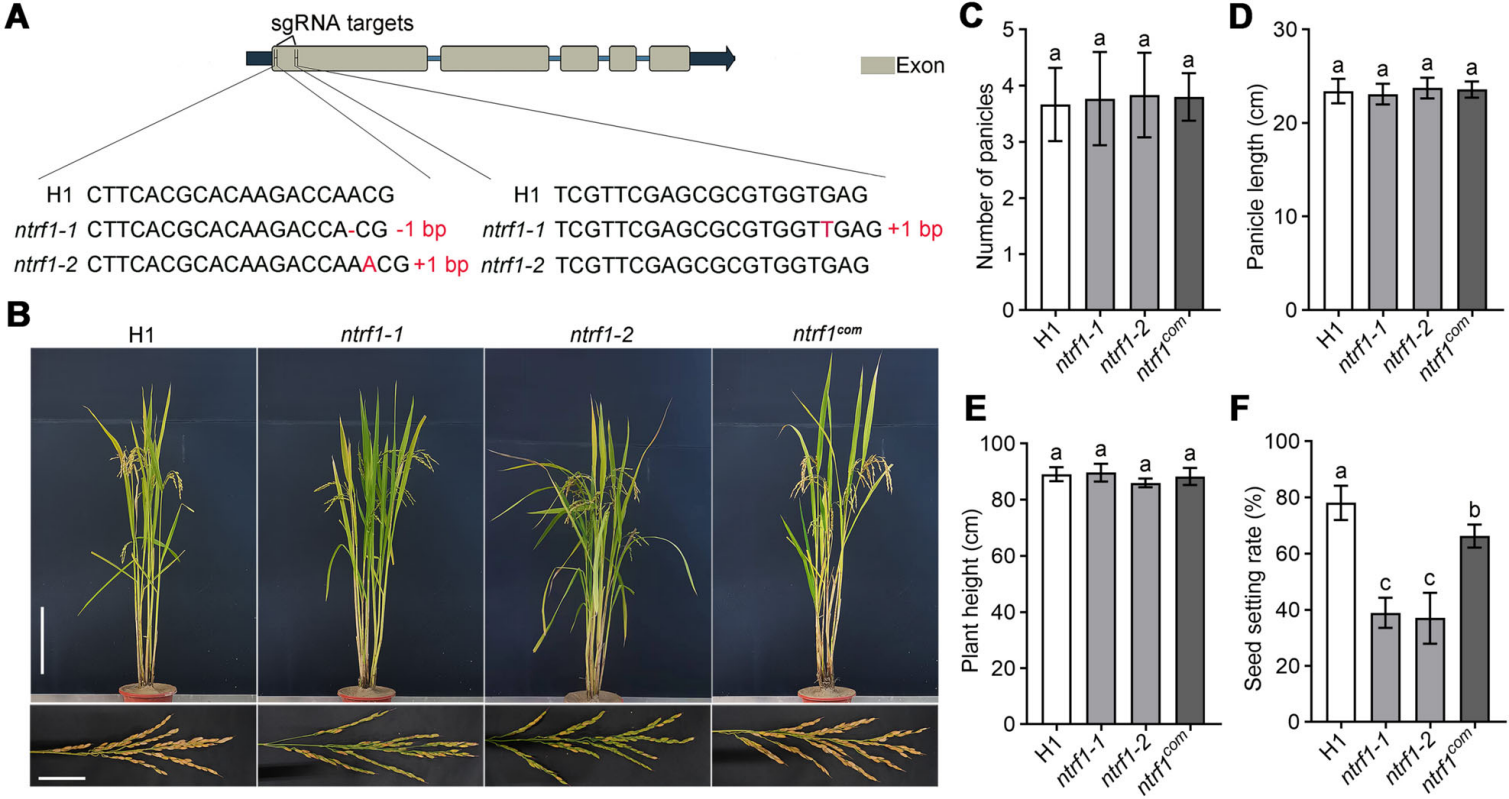
**Phenotypic characterization of** ***ntrf1*** **(A)** Schematic diagram of *NTRF1* gene editing. Two sgRNAs targeting *NTRF1* were designed, and two mutants, *ntrf1‐1* and *ntrf1‐2*, were identified. Exons are represented by gray rectangles, and non‐translated regions by black rectangles. The mutations are highlighted in red. **(B)** Plant morphology and mature panicles of Huaduo 1 (H1), *ntrf1* mutant (*ntrf1‐1* and *ntrf1‐2*), and *NTRF1* functional complementation line (*ntrf1* ^*com*^). The scale bars for the plant morphology and mature panicle diagram are 20 and 5 cm, respectively. **(C)** Number of panicles. **(D)** Panicle length. **(E)** Plant height. **(F)** Seed‐setting rate in H1, *ntrf1*, and *ntrf1* ^*com*^ lines. Data are presented as means ± *SD* (*n* = 15). Different letters indicate significant differences (*P* < 0.05; one‐way ANOVA, LSD test).

The *ntrf1* mutant was generated in our previous research using clustered regularly interspaced short palindromic repeats (CRISPR)/CRISPR‐associated protein 9 (Cas9)‐mediated gene editing technology in the Huaduo 1 (H1) background (Yu et al., 2021). In the present study, we employed Sanger sequencing analysis to identify two T‐DNA‐free homozygous frameshift mutants (designated *ntrf1‐1* and *ntrf1‐2*) in the T_4_ generation of the *ntrf1* mutant. Among the mutants, *ntrf1‐1* harbors a 1‐bp deletion at the first sgRNA position and a 1‐bp insertion at the second target site, while *ntrf1‐2* harbors a 1‐bp insertion at the first sgRNA position (Figures 1A, S1A–C). Frameshift position analysis showed that amino acid coding sequences terminate prematurely at 20 aa and 179 aa for *ntrf1‐1* and *ntrf1‐2*, respectively (Figure S1D). Phenotypic analysis confirmed that the *ntrf1* mutant showed no significant differences in the number of panicles, panicle length, and plant height compared to Huaduo 1 (H1, wild type) (Figure 1B–E). However, the seed‐setting rates of *ntrf1* mutants (38.92% and 36.99%) were significantly lower than that of H1 (78.10%) under normal conditions (Figure 1F). To verify the phenotype resulting from the loss of *NTRF1*, a functional complementation line (*ntrf1* ^*com*^) was constructed by introducing a 5,603‐bp genomic DNA fragment of *NTRF1* into the *ntrf1‐1* mutant (Figure S1E). The *ntrf1* ^*com*^ line exhibited a significantly restored seed‐setting rate of 66.30% compared to the *ntrf1* mutant (Figure 1F). These results indicate that *NTRF1* plays a crucial role in regulating the fertility of the NTR under normal conditions.

Since a specific SNP variation was found in *NTRF1* (Yu et al., 2021), the coding sequences (CDS) of *NTRF1* and *HSP101* were amplified from cDNA obtained from meiotic anthers of H1 (neo‐tetraploid rice) and Taichung 65 (T65, diploid rice) under normal conditions. Interestingly, amplification of the *NTRF1* CDS from cDNA of H1 yielded two transcripts: One fully spliced transcript with a length of 2,739 bp, and another transcript retaining all introns, with a length of 3,106 bp (Figure S2A). In contrast, *HSP101* transcripts were undetectable in diploid rice under normal conditions; a 2,739 bp transcript could only be amplified after heat shock treatment (Figure S2B). Furthermore, due to the non‐synonymous mutation resulting from a different SNP between NTRF1 and HSP101, AlphaFold2 was used to predict the tertiary structures of the proteins. The results showed that a new hydrogen bond was formed between the third alanine (Ala) and methionine (Met) residues in NTRF1 due to the mutation (Figure S2C). Additionally, this SNP also altered the restriction enzyme recognition site, changing from the *BspEI* site in *HSP101* to the *BsmI* site in *NTRF1* (Figure S2D, E).

In addition, in order to investigate whether disruption of the *HSP101* gene, which is located on the same genomic site as *NTRF1*, affects seed‐setting rate in diploid rice under normal conditions, we used the CRISPR/Cas9 gene editing system to knock out *HSP101* in the diploid rice, Taichung 65 (T65, *O. sativa* L. subsp. *japonica*) and Huanghuazhan (HHZ, *O. sativa* L. subsp. *indica*). In the T65 background, the wild type (WT) plants had a seed‐setting rate of 88.62%, while the two *hsp101* mutants exhibited comparable values of 87.97% and 86.02%, respectively. Similarly, in the HHZ background, the WT plants exhibited a seed‐setting rate of 86.85%, while the *hsp101* mutants recorded rates of 88.54% and 86.28%, respectively. In both genetic backgrounds of diploid rice, there were no significant differences in seed‐setting rates between the mutants and their WT plants (Figure S3A–C). Based on the above results, we consider that *HSP101* may not be necessary to regulate the fertility of diploid rice under normal conditions, and that the specific SNP variation (C to G) in NTR may be one of the important reasons for the neofunctionalization of the *NTRF1* gene.

### Mutation of *NTRF1* reduces pollen viability in neo‐tetraploid rice (NTR)

**Figure 2:**
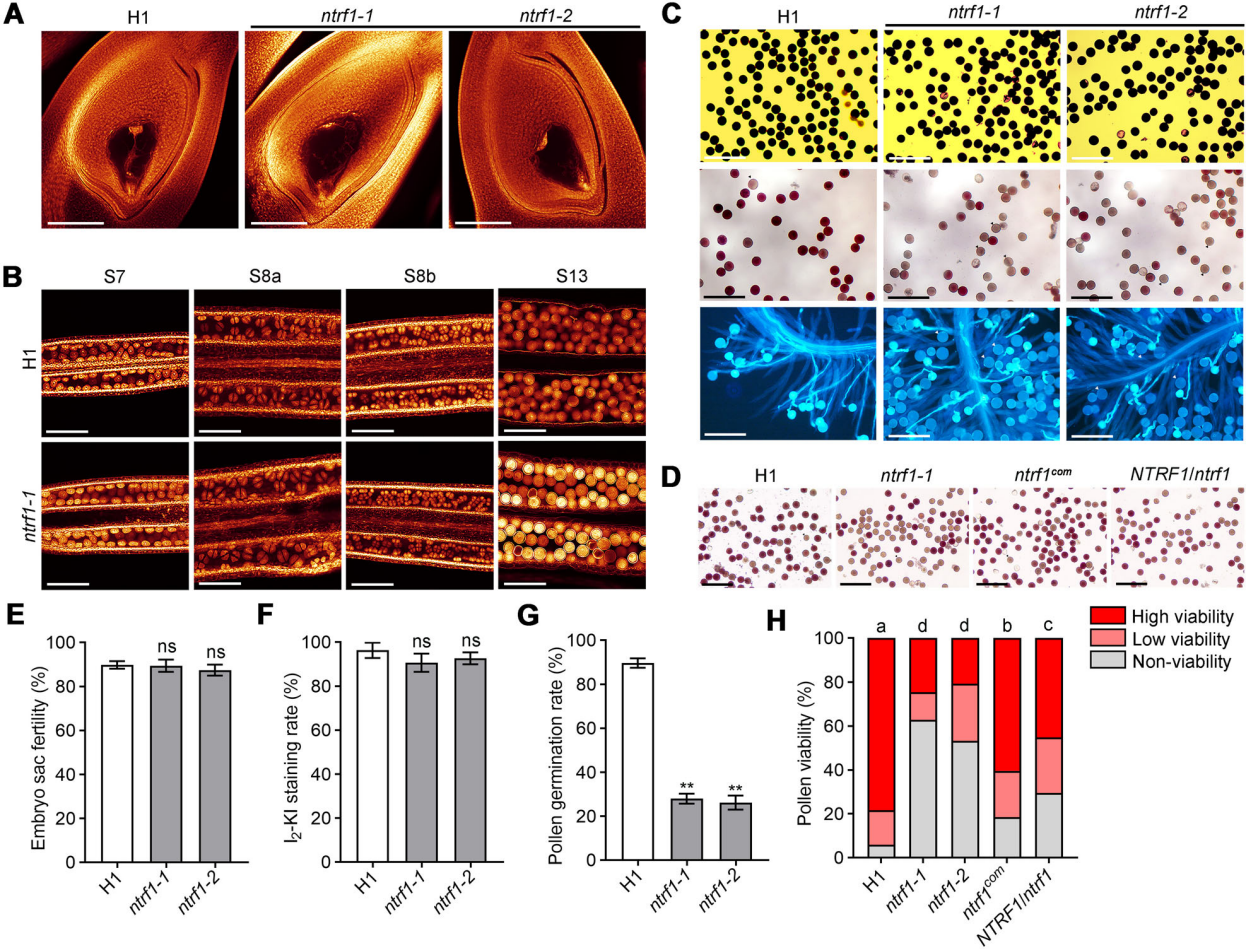
**Observation on the fertility of pollen and embryo sac in Huaduo 1 (H1) and** ***ntrf1*** **(A)** Observation of Huaduo 1 (H1) and *ntrf1* mutants' mature embryo sacs using Whole‐mount Eosin B‐staining Confocal Laser Scanning Microscopy (WE‐CLSM). Bars = 200 μm. **(B)** Observation of anthers at different developmental stages (S7, S8a, S8b, and S13) using WE‐CLSM. Bars = 200 μm. **(C)** I_2_‐KI staining of pollen grains, 1% 2, 3, 5‐Triphenyltetrazolium chloride (TTC) staining of pollen grains, and *in vivo* germination of pollen grains in H1 and *ntrf1* mutants. Bars = 200 μm. **(D)** 1% TTC staining of pollen grains in H1, *ntrf1* mutant, *ntrf1* ^*com*^ (*NTRF1* functional complementation line), and *NTRF1*/*ntrf1*. *NTRF1*/*ntrf1* indicated (*NTRF1* × *ntrf1*) F_1_ lines. Bars = 200 μm. The triangular‐shaped point to the abortive pollen grains. **(E**–**G)** Statistical results of embryo sac fertility, I_2_‐KI staining rate, and pollen germination rate in H1 and *ntrf1* mutants (means ± *SD*; *P < 0.05, **P < 0.01, ns, non‐significant; two‐tailed Student's *t*‐test; *n* = 30). **(H)** Pollen viability in H1, *ntrf1*, *ntrf1* ^*com*^, and F_1_ lines. Different letters indicate significant differences (*P* < 0.05; one‐way ANOVA, LSD test; *n* = 30).

To explore the cause of the reduced seed‐setting rate in *ntrf1*, a systematic comparative analysis was performed between H1 and the *ntrf1* mutant, focusing on mature embryo sac development, pollen stainability, pollen germination, and pollen viability. Whole‐mount Eosin B‐staining Confocal Laser Scanning Microscopy (WE‐CLSM) analysis confirmed that embryo sac fertility in *ntrf1* (89.41% and 87.45%) did not differ significantly from that in H1 (control, 89.82%) (Figure 2A, E). Furthermore, compared to H1, no significant morphological abnormalities were observed in *ntrf1* pollen at stages 7, 8, and 13 (S7, S8, and S13) (Figure 2B). Anther developmental stages were classified according to established morphological criteria (Zhang et al., 2011). I_2_‐KI staining presented no significant difference in pollen staining rate between the *ntrf1* mutant (86.34% and 91.22%) and H1 (94.98%) (Figure 2C, F).

However, the pollen germination rates of the *ntrf1* mutant (27.97% and 26.18%) on the stigma 30 min after flowering were significantly lower than H1 (89.69%), as determined by sodium hydroxide transparency technology (Figure 2G). Additionally, staining with a 1% 2, 3, 5‐Triphenyltetrazolium chloride (TTC) solution to assess pollen viability illustrated that 78.39% of the pollen in H1 turned a dark red color (indicating high viability), while only 5.93% appeared gray (indicating non‐viability). In contrast, *ntrf1* pollen displayed significantly reduced viability, with merely 24.56% and 20.78% of grains stained dark red, while non‐viable pollen accounted for 62.77% and 53.33%, respectively (Figure 2C, H). To understand the mode of action of *NTRF1*, TTC staining was performed, and the results showed that pollen viability in the *NTRF1*/*ntrf1* lines (45.20%) was intermediate between the H1 (78.39%) and the *ntrf1* mutants (24.56% and 20.78%). This result suggested that *NTRF1* might function in a gametophytic manner. Furthermore, the pollen viability in the *ntrf1* ^*com*^ line was significantly restored to 60.36% (Figure 2D, H). These results signified that impaired pollen viability was the primary reason for the reduced seed‐setting rate in the *ntrf1* mutant.

### *NTRF1* affects pollen starch synthesis and mitochondrial development in neo‐tetraploid rice (NTR)

**Figure 3:**
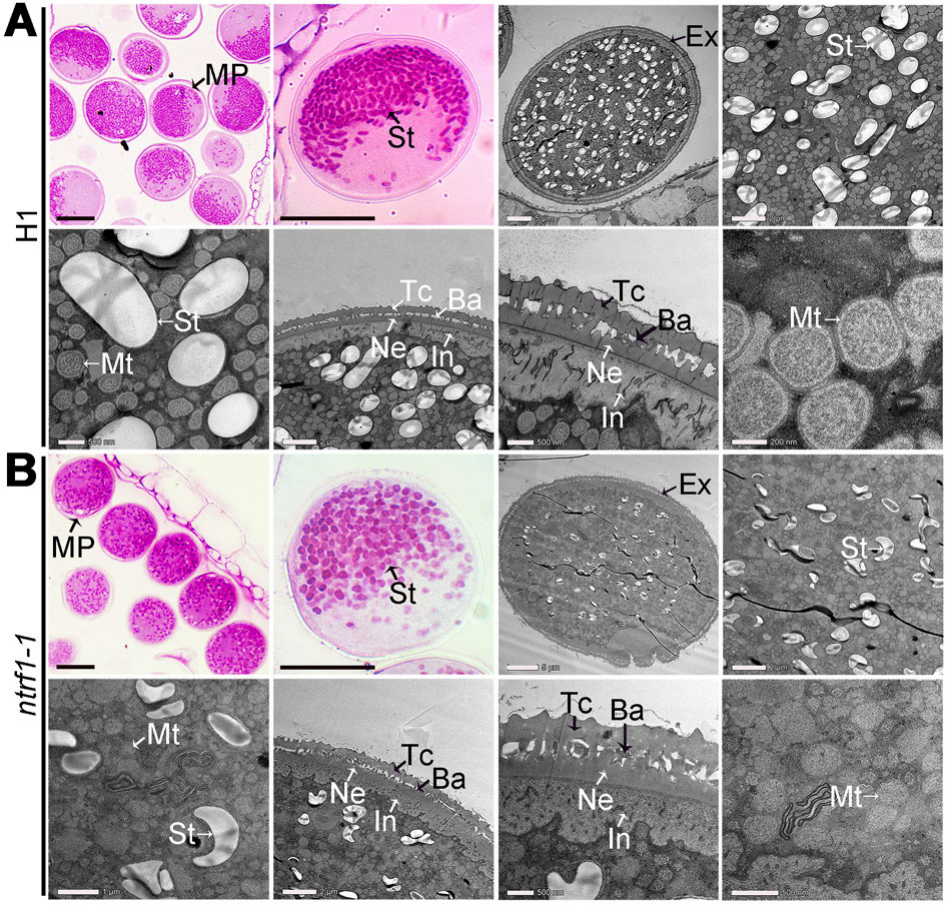
**Cytological observation of mature pollen grains in Huaduo 1 (H1) and** ***ntrf1*** **(A)** Semi‐thin sections of mature anthers from H1, stained with periodic acid‐Schiff (PAS) and observed by an optical microscope. Bars = 30 μm. Ultrastructure of mature pollen grains from H1, as observed by transmission electron microscopy (TEM). The scale bars appear in the lower left corner of each TEM image. **(B)** Semi‐thin sections of mature anthers from *ntrf1*, stained with PAS. Bars = 30 μm. Ultrastructure of mature pollen grains from *ntrf1*, observed by TEM. The scale bars appear in the lower left corner of each TEM image. Ba, bacula; Ex, exine; In, intine; MP, mature pollen; Mt, mitochondrion; Ne, nexine; St, starch grain; Tc, tectum.

Since *NTRF1* knockout results in reduced pollen viability, the structural features of mature pollen in the *ntrf1‐1* mutant warrant investigation. Pollen grains were analyzed using periodic acid‐Schiff (PAS) staining and transmission electron microscopy (TEM). PAS staining presented that starch granules in H1 pollen exhibited a typical densely packed arrangement (Figure 3A). TEM observation indicated that H1 pollen grains displayed a well‐defined morphology with fully developed, intact starch granules. The exine outer layer exhibited smooth contours, the columellate layer maintained uniform thickness, and the exine‐intine interface was discernible. Additionally, spherical mitochondria with well‐defined structures were abundantly distributed within H1 pollen (Figure 3A).

In contrast, *ntrf1‐1* pollen showed a looser organization and significantly reduced starch accumulation (Figure 3B). The *ntrf1‐1* mutant exhibited underdeveloped starch granules, most of which appeared crescent‐shaped. The boundary between the pollen exine inner layer and the intine was indistinct, and the thickness of the columellate layer was uneven (Figure 3B). Furthermore, the mitochondrial structure appeared blurred, and its integrity was compromised. Scanning electron microscopy (SEM) further confirmed that *ntrf1‐1* pollen grains were slightly smaller in volume compared to H1 (Figure S4A, B). Quantification of pollen grain radii using ImageJ software, followed by volume calculation, validated a significant reduction in the pollen grain volume of the *ntrf1‐1* mutant (40,995.32 μm³) compared to H1 (44,737.16 μm³) (Figure S4C).

### *NTRF1* is required for tapetal programmed cell death (PCD) in neo‐tetraploid rice (NTR)

**Figure 4:**
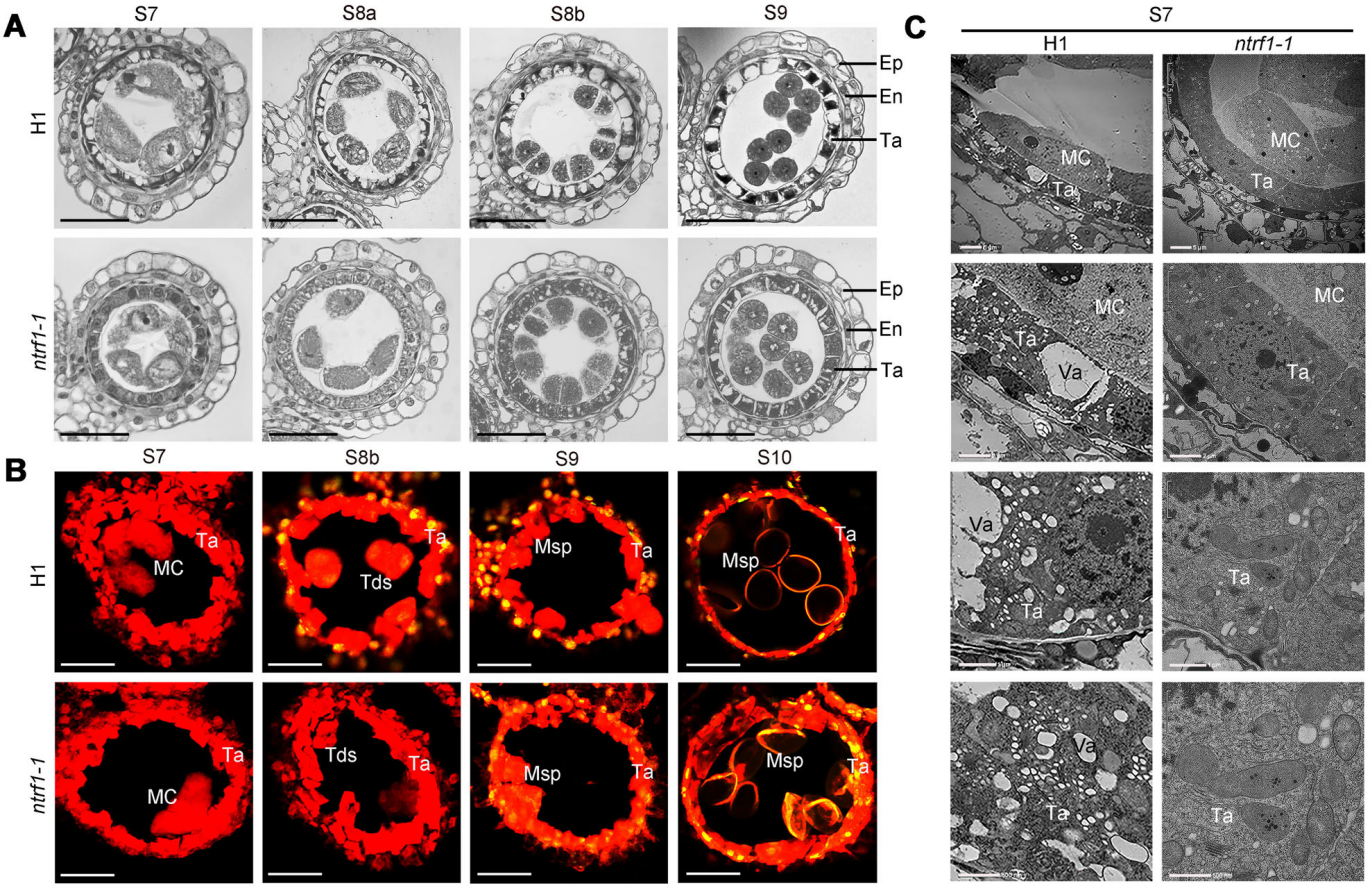
**Analysis of programmed cell death (PCD) in anthers of Huaduo 1 (H1) and** ***ntrf1*** **(A)** Semi‐thin section analysis of anther development at the S7 to S9 stages in H1 and *ntrf1*, respectively. Bars = 50 μm. **(B)** Terminal deoxynucleotidyl transferase‐mediated dUTP nick‐end labeling (TUNEL) staining characteristics of anther development at the S7 to S10 stages in H1 and *ntrf1*, respectively. Bars = 100 μm. **(C)** Transmission electron microscopy (TEM) observations of tapetum layer development at S7 in H1 and *ntrf1*, respectively. The scale bars in the figure, from top to bottom, are 5 μm, 2 μm, 1 μm, and 500 nm, respectively. They also appear in the lower left corner of each image. En, endothecium; Ep, epidermis; MC, meiocyte; Msp, microspore; Ta, tapetum layer; Tds, tetrads; Va, vacuole.

To investigate the cytological mechanism of pollen abortion in *ntrf1* mutants, semi‐thin sections of anthers from H1 and the *ntrf1‐1* mutant were examined. In H1 anthers, pollen mother cells (PMCs) assumed a circular arrangement along the tapetum during stages S7 to S8b (S7, S8a, and S8b). Concurrently, tapetal cells underwent vacuolization and exhibited typical PCD characteristics (Figure 4A). By stage 9 (S9), their degradation continued alongside cytoplasmic condensation. During stages S10–S11, the tapetum degraded into cellular debris and vacuolated microspores underwent the first mitosis. Subsequently, the tapetum completely disappeared by stages S12–S13 (Figure S5A). In contrast, tapetal cells of the *ntrf1‐1* mutant from stages S7 to S8b exhibited abundant cytoplasm and no obvious vacuolation, suggesting a delay in PCD initiation. The tapetum in the *ntrf1‐1* mutant initiated vacuolation at stage S9 (Figure 4A), showed deeper staining compared to H1 during stages S10–S11, and nearly completely disappeared at the S12–S13 stage (Figure S5A).

To elucidate the mechanism underlying delayed tapetum degradation in *ntrf1‐1*, a terminal deoxynucleotidyl transferase‐mediated dUTP nick‐end labeling (TUNEL) assay was employed to monitor PCD progression from S7 to S13. In H1, apoptotic signals were detected in the tapetum at S8–S10, whereas the apoptotic signals in the *ntrf1‐1* mutant were delayed until stage S9 (Figure 4B). During the S11 to S13 stages, apoptotic signals were not observed in H1 and the *ntrf1‐1* mutant (Figure S5B).

TEM analysis confirmed that there were no significant differences in anther structure between H1 and *ntrf1‐1* at S6 (Figure S5C). During the S7 stage, the PMCs in both H1 and *ntrf1‐1* mutants began to approach the tapetum. At this stage, numerous small vesicles were observed around the nucleus of tapetal cells in H1, while large vacuoles formed in the peripheral region. Most organelles were severely degraded, and the cytoplasm was condensed. Conversely, the tapetal cells of the *ntrf1‐1* mutant were tightly arranged and contained visible organelles, such as mitochondria and Golgi bodies, with intact nuclear envelopes (Figure 4C). Taken together, these findings demonstrated that *NTRF1* was an important regulator of tapetal PCD and that the loss of its function delayed PCD initiation.

### *NTRF1* impacts the expression of ROS and ABA‐related genes

**Figure 5:**
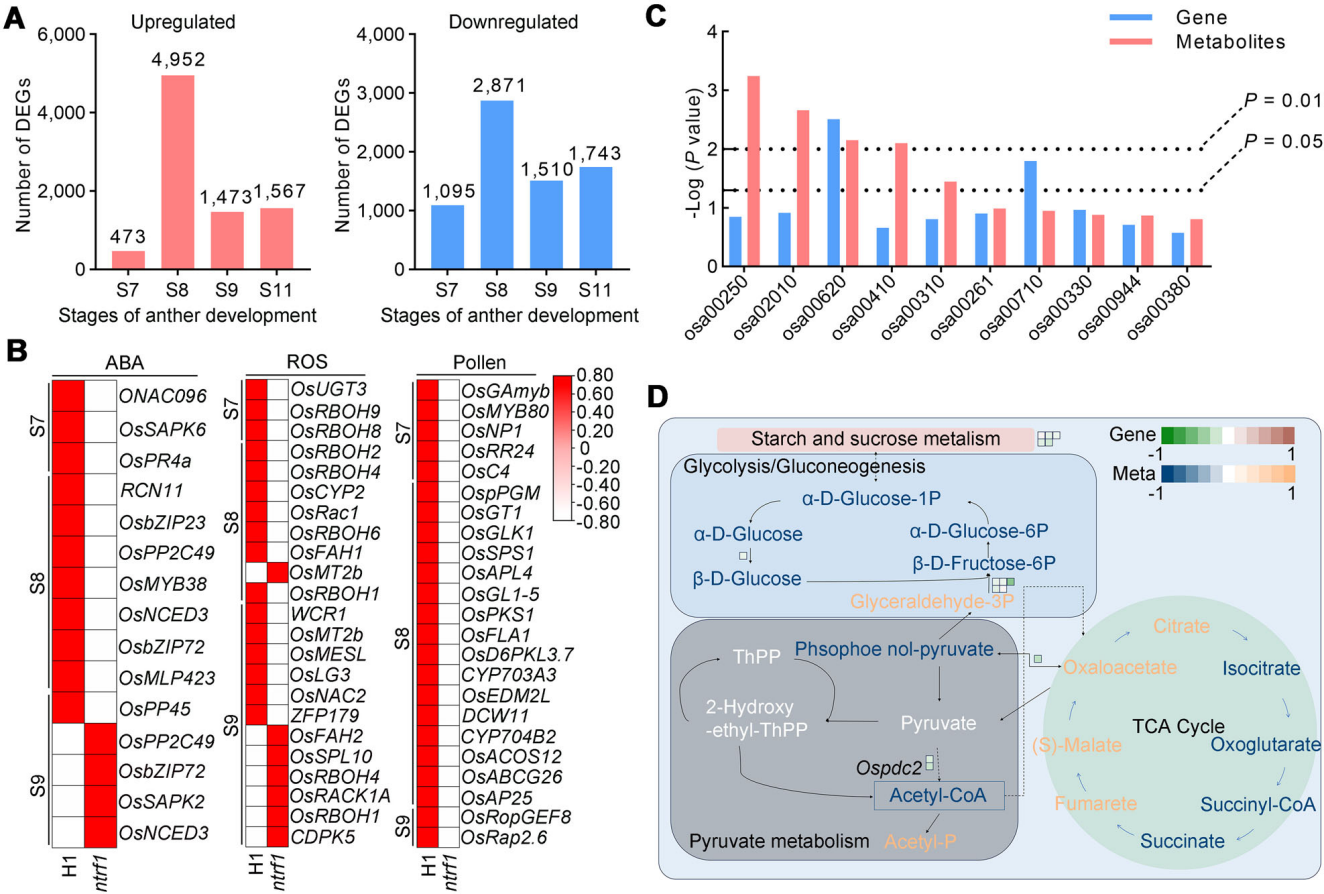
**Transcriptome and metabolome analyses of anthers at different developmental stages in Huaduo 1 (H1) and** ***ntrf1*** **(A)** Analysis of differentially expressed genes (DEGs) in H1 and *ntrf1* at the S7, S8, S9, and S11 stages. **(B)** Analysis of DEGs related to ABA, ROS, and pollen development at the S7, S8, and S9 stages, respectively. The heatmap displays the Log_2_ (TPM) values of DEGs. **(C)** Kyoto Encyclopedia of Genes and Genomes (KEGG) analysis showing significant differences in the pyruvate metabolism pathway (osa00620) in both S11's transcriptome and S12's metabolome of H1 and *ntrf1‐1* mutant (*P* < 0.01). **(D)** Alterations in the accumulation levels of key metabolites and transcriptional abundances of pivotal genes within the glycolytic pathway and tricarboxylic acid (TCA) cycle.

RNA‐seq analysis was performed on anther tissues from the *ntrf1‐1* mutant and H1 at four developmental stages (S7, S8, S9, and S11). Quality assessment of the sequencing data confirmed that the percentage of Q30 bases (sequencing accuracy of 99.9%) exceeded 92.43% across all samples. Alignment rates to the MSU7 (Nipponbare, *O*. *sativa japonica*) reference genome ranged from 93.54% to 95.79% (Table S1). Analysis of differentially expressed genes (DEGs) identified 473, 4,952, 1,473, and 1,567 upregulated genes, along with 1,095, 2,871, 1,510, and 1,743 downregulated genes across the four stages, respectively (Figure 5A).

Given the close association between the initiation of tapetal PCD and ROS accumulation (Yi et al., 2016; Zhao et al., 2023; Chen et al., 2023b), DEGs related to ROS metabolism during the S7 to S9 stages were specifically analyzed. The results showed that ROS‐generating genes were downregulated in *ntrf1‐1* at S7 and S8. The downregulation of NADPH oxidase genes is particularly significant: *OsRBOH8* and *OsRBOH9* at S7; *OsRBOH1*, *OsRBOH2*, *OsRBOH4*, and *OsRBOH6* at S8. Conversely, the ROS‐scavenging gene *OsMT2b* was upregulated at S8. Other ROS‐generating genes, such as *OsUGT3*, *OsFAH1*, and *OsCYP2*, also showed downregulation. Since ROS generation was induced by ABA signaling, genes associated with ABA biosynthesis and signal transduction were examined and found to be downregulated, including *OsSAPK6*, *OsbZIP23*, *OsPP2C49*, *OsNCED3*, and *OsbZIP72* (Figure 5B).

However, an opposite expression trend for ROS and ABA‐related genes was observed at S9. ROS‐generating genes (*OsFAH2*, *OsRBOH1*, *OsRBOH4*, *CDPK5*) and ABA signaling/biosynthesis genes (*OsPP2C49*, *OsbZIP72*, *SAPK2*, *OsNCED3*) were significantly upregulated (Figure 5B). Subsequently, RT‐qPCR was employed to validate the differential expression of ABA‐ and ROS‐related genes, confirming the reliability of RNA‐seq data (Figures S6, S7). These results indicated that the delayed tapetal PCD in the *ntrf1* mutant may be modulated by ABA and ROS signaling molecules.

### *NTRF1* influences the expression of genes involved in pollen development

To further clarify the role of *NTRF1* in pollen development, genes associated with anther development among the DEGs were analyzed. Kyoto Encyclopedia of Genes and Genomes (KEGG) enrichment analysis revealed that downregulated DEGs were enriched in metabolic pathways critical for pollen development, including fatty acid biosynthesis, cutin/suberine/wax biosynthesis, and starch and sucrose metabolism (Figure S8A–D). Additionally, among the downregulated DEGs from stages S7 to S9, multiple genes involved in pollen development were identified. These included tapetum development genes (*OsGAmyb*, *OsMYB80*, *OsAP25*, *OsRR24*, *OsC4*, *OsEDM2L*), pollen wall development genes (*OsNP1*, *OsPKS1*, *CYP703A3*, *CYP704B2*, *OsACOS12*), and genes related to nutrient synthesis within pollen (*OspPGM*, *OsSPS1*, *OsAPL4*) (Figure 5B). Subsequently, RT‐qPCR analysis further verified the reliability of RNA‐seq results (Figure S9).

To investigate the metabolic basis underlying the decline in pollen viability during late development, untargeted metabolomic analysis was conducted on S12 anthers. The results showed that a total of 287 metabolites were identified. Based on the criteria of *P* < 0.05 and variable importance for the projection (VIP) > 1, 93 differentially accumulated metabolites were identified, of which 64 were upregulated and 29 were downregulated (Figure S10A, B). To explore the correlation between gene expression changes and differential metabolite accumulation during the late stage of pollen development in H1 and *ntrf1‐1* mutants, an integrated analysis of the S11's transcriptomic and S12's metabolomic datasets was conducted. This analysis revealed that the pyruvate metabolism pathway (osa00620) was significantly enriched (*P* < 0.01) in the KEGG pathways of both datasets (Figures 5C, S10C). Given the established link between pyruvate metabolism and ATP generation (Karikomi et al., 2024), we observed a significant reduction in acetyl‐CoA, a key intermediate in glycolysis and the tricarboxylic acid (TCA) cycle. Concomitantly, the expression of LOC_Os03g18220 (*Ospdc2*, encoding pyruvate decarboxylase) was downregulated (Figure 5D). These results show that *NTRF1* mutation affects the expression of key pollen development genes and disrupts pollen energy metabolism, leading to reduced pollen viability.

### NTRF1 interacts with SAPK2, a positive regulator of ABA signaling

**Figure 6:**
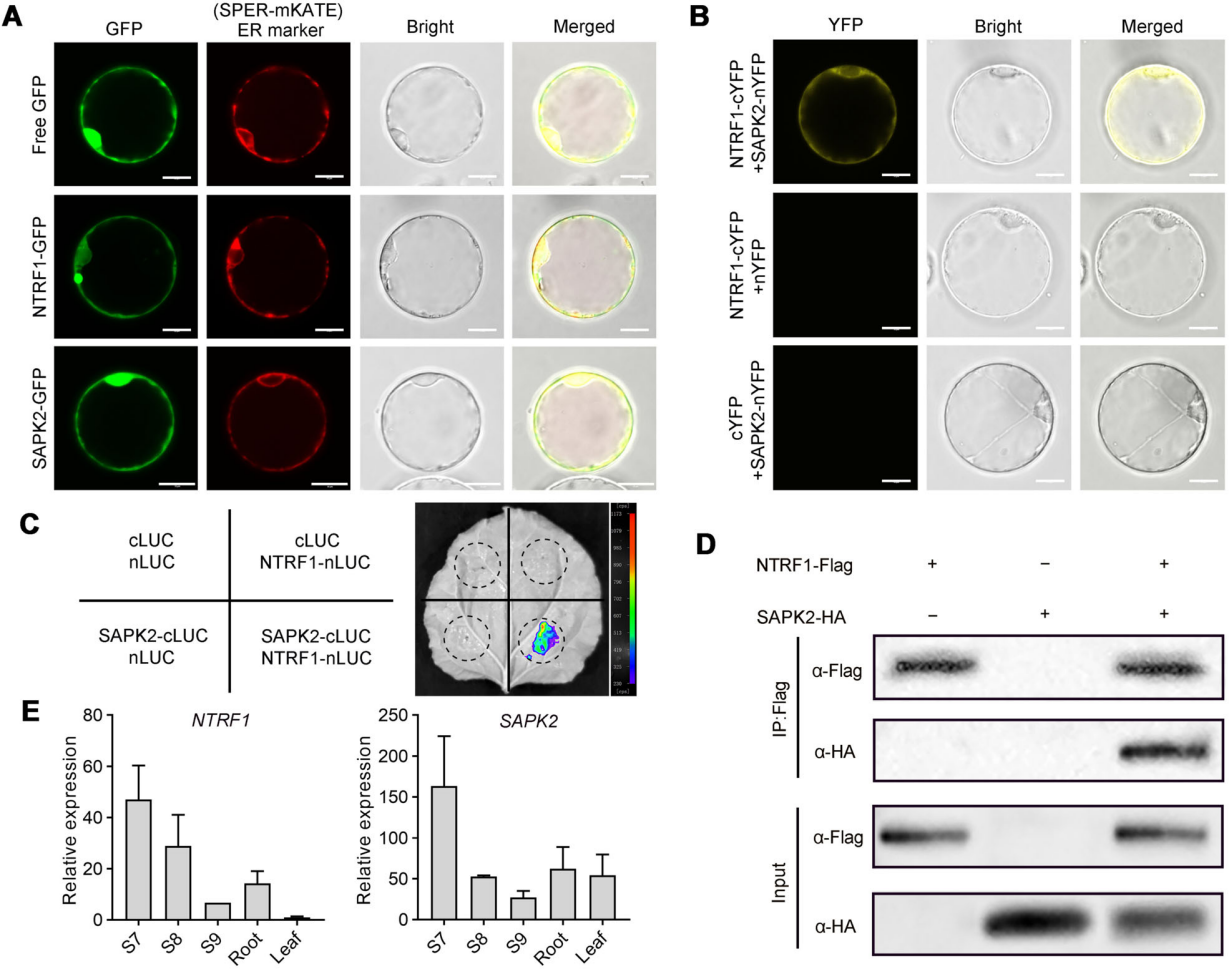
Physical interaction between NTRF1 and SAPK2 **(A)** Subcellular localization of NTRF1‐GFP and SAPK2‐GFP fusion proteins in rice protoplasts. SPER‐mKATE was used as an ER marker. Bars = 10 μm. **(B)** Bimolecular fluorescence complementation (BiFC) assay demonstrating the interaction between NTRF1 and SAPK2 in rice protoplasts. Bars = 10 μm. **(C)** Luciferase complementation imaging (LCI) assay showing interaction between NTRF1 and SAPK2 in epidermal cells of *Nicotiana benthamiana* leaves. **(D)** Co‐immunoprecipitation (Co‐IP) assay showing the interaction between NTRF1 and SAPK2 *in vivo*. NTRF1‐Flag was immunoprecipitated using anti‐Flag affinity magnetic beads, and co‐precipitated SAPK2‐HA was detected using an anti‐HA antibody. **(E)** Expression patterns of *NTRF1* and *SAPK2* were analyzed by RT‐qPCR in anthers at the S7–S9 stages, root and leaf of H1.

To clarify the potential mechanism by which *NTRF1* affects pollen development, a yeast two‐hybrid (Y2H) assay was performed to identify interacting proteins. The results showed that NTRF1 interacted with stress‐activated protein kinase 2 (SAPK2) *in vitro*, and NTRF1 had auto‐activation activity in the membrane yeast two‐hybrid system (Figure S11). As SAPK2 was a known positive regulator of ABA signaling (Zong et al., 2016), we focused on SAPK2 for further investigation. Subcellular localization analysis confirmed that NTRF1 was localized to the cytoplasm, while SAPK2 was localized to both the cytoplasm and the nucleus (Figure 6A). The interaction between NTRF1 and SAPK2 was substantiated using bimolecular fluorescence complementation (BiFC). Fluorescence signals from NTRF1‐cYFP and SAPK2‐nYFP were predominantly localized in the cytoplasm, consistent with their cytoplasmic localization (Figure 6B). To further corroborate their interaction, luciferase complementation imaging (LCI) and co‐immunoprecipitation (Co‐IP) assays were conducted in *Nicotiana benthamiana;* these assays confirmed the interaction between NTRF1 and SAPK2 (Figure 6C, D). The above results demonstrated that NTRF1 physically interacted with SAPK2 both *in vitro* and *in vivo*. RT‐qPCR analysis showed that the expression patterns of *NTRF1* and *SAPK2* in early anther tissues were highly similar, with transcript levels reaching a peak at S7 (Figure 6E), suggesting potential co‐regulation or functional cooperation.

### Exogenous application of ABA partially restores the fertility in the *ntrf1* mutant

**Figure 7:**
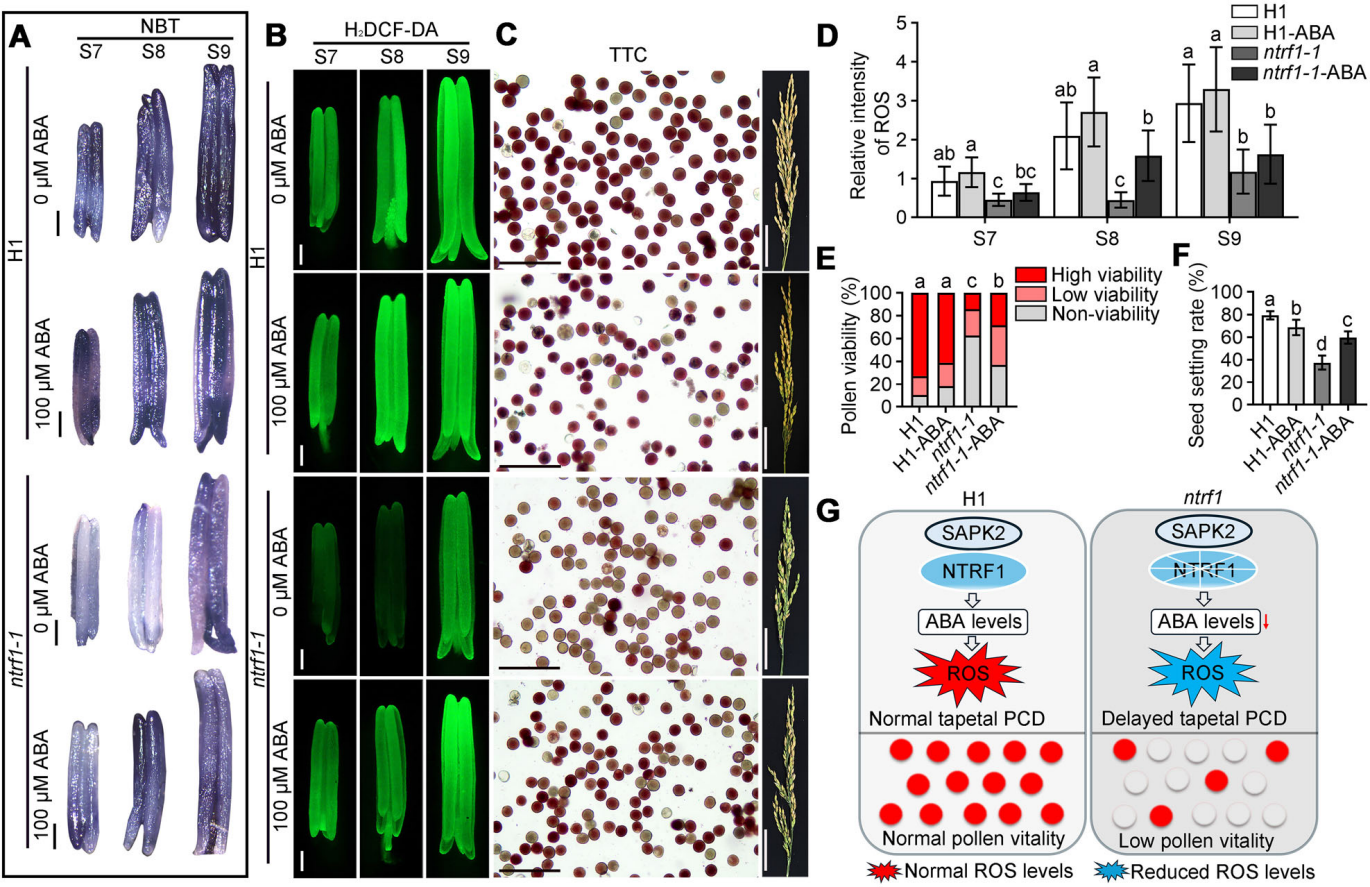
**Reactive oxygen species (ROS) analysis in anthers of Huaduo1 (H1) and** ***ntrf1*** **under exogenous abscisic acid (ABA) treatment** **(A)** Nitroblue tetrazolium (NBT) staining of superoxide anion production in anthers of H1 and *ntrf1‐1* at S7–S9 stages, with or without ABA treatment. Bars = 250 μm. **(B)** Analysis of ROS using 2’,7’‐dichlorodihydrofluorescein diacetate (H_2_DCF‐DA) in H1 and *ntrf1‐1* anthers at S7–S9 stages, with or without ABA treatment. Bars = 250 μm. **(C)** 1% TTC staining of pollen grains and panicle observation. The scale bars for TTC and panicle morphology diagrams are 200 μm and 5 cm, respectively. **(D)** Relative quantification of ROS levels based on H_2_DCF‐DA fluorescence intensity in anthers at S7–S9 stages (means ± *SD*; *n* = ~90). **(E)** Statistical analysis of mature pollen viability (means ± *SD*; *n* = 25). **(F)** Statistical analysis of seed‐setting rates (means ± *SD*; *n* = 10). **(D**–**F)** Different letters indicate significant differences (*P* < 0.05; one‐way ANOVA, LSD test). **(G)** A working model of NTRF1‐SAPK2 interaction regulating pollen development in NTR. NTRF1 regulates ROS production by interacting with SAPK2. Normal ROS levels will promptly induce PCD in the tapetum, ensuring the formation of highly active pollen. In the *ntrf1* mutant, ABA and ROS levels decrease, leading to pollen partial sterility.

ABA plays a critical role in regulating pollen viability by modulating ROS levels and inducing PCD in tapetal cells (Zhao et al., 2023). To investigate whether disrupted ROS dynamics contribute to the anther developmental defects observed in the *ntrf1* mutant. Firstly, nitroblue tetrazolium (NBT) staining was performed on anthers, and the results showed that ROS significantly accumulated in H1 anthers during S7 to S9, while ROS accumulation was notably reduced in *ntrf1‐1* anthers. This result was further validated using the fluorescent probe 2’,7’‐dichlorodihydrofluorescein diacetate (H_2_DCF‐DA). Distinct ROS signals were detected in H1 anthers from S7 to S9, whereas signals in *ntrf1‐1* were nearly undetectable during S7 to S8 (Figure 7A, B). Quantitative analysis of the fluorescence intensity validated that ROS levels in H1 anthers were significantly higher than *ntrf1‐1* during S7 to S9 (Figure 7D). Consistent with this, quantification of endogenous ABA levels in meiotic‐stage anthers confirmed a significant reduction in the *ntrf1‐1* mutant (11.99 ng/g) compared to H1 (14.47 ng/g) (Figure S12).

Furthermore, at the S8 stage, treatment with 100 μM ABA during the booting stage did not significantly change ROS levels in the anthers of H1, but it led to a significant increase in *ntrf1‐1*. More importantly, exogenous ABA treatment rescued the reproductive developmental defects observed in the *ntrf1‐1* mutant (Figure 7A–D). Statistical analysis demonstrated that ABA application decreased the aborted pollen percentage from 62.78% to 37.03% (11.80% in H1 and 18.08% in the ABA‐treated H1) and improved the seed‐setting rate from 37.38% to 59.71% (79.32% in H1). The seed‐setting rate of ABA‐treated H1 was 68.59%, which was slightly lower than that of untreated H1 (Figure 7E, F). Collectively, these findings demonstrate that *NTRF1*‐mediated regulation of ROS homeostasis is essential for normal pollen development (Figure 7G).

## DISCUSSION

### *NTRF1* is essential for pollen development in neo‐tetraploid rice (NTR)

The NTR was novel elite germplasm that could be utilized in polyploidy breeding in rice, and some polyploidy hybrid rice with high yield had been developed using the germplasm (Ghaleb et al., 2020; Liu et al., 2023). In this study, a critical role for the *HSP101* allelic variant *NTRF1* in pollen development of NTR was identified. The *NTRF1* mutation significantly reduced the seed‐setting rates, which was caused by low pollen viability. Cytological studies showed that impaired pollen viability may have resulted from delayed tapetal PCD in the *ntrf1* mutant. The *ntrf1* mutants exhibited abnormal starch grains and mitochondrial morphologies of mature pollen. Moreover, the *ntrf1* mutants also showed disrupted pyruvate metabolism. Given pyruvate's role in ATP production via the TCA cycle (Liu et al., 2022b), these perturbations likely contributed to energy deficits that affected pollen germination (Chen et al., 2023c; Shi et al., 2024b). These results indicated that *NTRF1* plays a crucial role in tapetal PCD. Delayed progression of PCD disrupts nutritional support for microspore development, leading to reduced pollen viability and impaired germination.

In the present study, we also found that *NTRF1* mutation could affect the expression of tapetum genes, such as *OsGAmyb*, *CYP703A3*, and *OsABCG26*. These genes have been found to play an important role in pollen development. *OsGAmyb*, a transcription factor, exhibits high expression specificity in anther tapetal cells, functioning upstream of the tapetal degradation regulator *TDR* (Liu et al., 2010). *CYP703A3*, a cytochrome P450 hydroxylase, is critical for exine formation (Yang et al., 2014). *OsABCG26* is a member of the ABC transporter family and is primarily involved in transporting lipid molecules within tapetum cells (Zhao et al., 2015). Meanwhile, expression of the starch and sucrose synthesis genes *OsGBSSI*, *OspPGM*, and *OsSPS1* was downregulated in the mutant. *OsGBSSI* controlled amylose content in pollen (Huang et al., 2021; Zhang et al., 2021). *OspPGM* is essential for plastidial starch synthesis (Lee et al., 2016), and *OsSPS1*‐mediated sucrose production is crucial for pollen germination (Hirose et al., 2014). The downregulation of these genes further clarified the mechanism of pollen abortion in *ntrf1* mutants.

Notably, under normal growth conditions, no significant difference in seed‐setting rate was found between the *hsp101* mutant and wild‐type diploid rice in this study, which was consistent with recent research (Li et al., 2025). As for why *NTRF1* defects, which is located at the same locus site as *HSP101*, could affect pollen development in NTR. We hypothesized that this phenotypic inconsistency may be linked to a unique SNP locus within *NTRF1*. This may influence the expression patterns of *NTRF1* in the reproductive tissues of NTR (Yu et al., 2021). Under normal conditions, amplification of the *NTRF1* CDS from cDNA of H1 meiotic‐stage anther*s* could generate two transcripts. In contrast, no transcript was detected in diploid rice under the same conditions. In addition, due to SNP variation, the third proline (Pro) in HSP101 was replaced by Ala in NTRF1, resulting in the formation of a new hydrogen bond between Met and Ala in NTRF1. This SNP variation also modified the restriction enzyme recognition site. The mechanism study of *NTRF1* has deepened our understanding of fertility regulatory networks. Future investigations will focus on the generation mechanism and the biological significance of this SNP.

### *NTRF1*‐mediated ROS homeostasis is indispensable for proper PCD progression

The anther wall consists of four somatic cell layers arranged from outermost to innermost: epidermis, endothecium, middle layer, and tapetum. As the innermost layer directly adjacent to microspores, the tapetum undergoes precisely regulated PCD during development (Yao et al., 2022). Accumulation of ROS has been established as a pivotal trigger for initiating tapetal PCD (Zhang et al., 2023; Singh et al., 2024). Our study demonstrated that the *NTRF1* mutation delayed tapetal PCD progression, which was associated with significantly reduced ROS levels during S7 to S9, consistent with established regulatory mechanisms (Chen et al., 2023b; Zhou et al., 2024). Furthermore, the ABA application could effectively restore ROS production and rescue the mutant phenotype. This suggests that *NTRF1* regulates ROS production by participating in the ABA pathway.

In this study, we found that genes involved in ABA synthesis and signaling (*OsNCED3*, *OsbZIP23*) were downregulated in the S7–S8 stages of the *ntrf1* mutant. *OsNCED3* encodes a 9‐cis‐epoxycarotenoid dioxygenase, a rate‐limiting enzyme in ABA biosynthesis (Chen et al., 2023d). OsbZIP23 is a key ABA‐responsive transcription factor, and its mutants show decreased ABA sensitivity (Zong et al., 2016). Additionally, the expression of ROS‐generating genes was altered, particularly NADPH oxidase genes (*OsRBOH1*, *OsRBOH8*), which played crucial roles in ROS generation (Wong et al., 2008; Shi et al., 2020). Previous studies had linked ROS accumulation to *OsDMI3* activation in ABA signaling, which may be modulated by *OsRBOH* genes (Wang et al., 2023). Furthermore, protein interaction assays confirmed that NTRF1 physically associates with SAPK2, an SnRK2‐type protein kinase that positively regulates ABA signaling (Kim et al., 2012; Lou et al., 2017). Notably, *SAPK2* knockdown impairs ABA‐mediated ROS generation and PCD initiation (Zhao et al., 2023), which mirrors the phenotype of the *ntrf1* mutant.

Interestingly, during the S9 stage in anthers, the expression levels of ABA biosynthesis and signaling genes (*OsNCED3*, *SAPK2*) as well as the ROS‐generating gene *OsRBOH1* were upregulated in the *ntrf1‐1* mutant, while the ROS‐scavenging gene *OsMT2b* exhibited an opposite expression trend compared to that in the S8 stage. It was postulated that ABA signal transduction may no longer depend on *NTRF1* during this developmental phase. This transition indicates that *NTRF1* has stage‐specific regulatory effects, which may be associated with its dynamic expression pattern in NTR anthers (Yu et al., 2021). Specialized regulatory mechanisms are believed to have evolved in polyploid rice to help mitigate the stress resulting from genome duplication (Zhang et al., 2015). In summary, we propose a working model for regulating NTR pollen development through the interaction between NTRF1‐SAPK2. In this model, NTRF1 regulates ROS production by interacting with SAPK2. Normal ROS levels will promptly induce PCD in the tapetum, ensuring the formation of highly active pollen. When *NTRF1* is deficient, ABA and ROS levels decrease, leading to pollen partial sterility (Figure 7G). Our studies provide new insight into the role of HSPs in ABA‐mediated ROS homeostasis and PCD regulation.

## MATERIALS AND METHODS

### Plant materials

The *ntrf1* mutant was constructed in our previous research (Yu et al., 2021) using the CRISPR/Cas9 system in the neo‐tetraploid rice (NTR) background, Huaduo 1 (H1). H1 was developed from the hybrid F_6_ of a cross between Jackson‐4x (ATR) and 96025 by our team in 2009 (Liu et al., 2023). The *hsp101* mutant was generated in two varieties of diploid rice, Taichung 65 (*japonica*) and Huanghuazhan (*indica*), using the same gene editing system (Yu et al., 2021). Transgenic complementary lines (*ntrf1* ^*com*^) were generated in the *ntrf1‐1* background. All materials were planted at the Teaching and Research Farm of South China Agricultural University in Guangdong, China.

### Cytological observation

The embryo sacs and anthers of all materials were observed using the Whole‐mount Eosin B‐staining Confocal Laser Scanning Microscopy (WE‐CLSM), with samples prepared according to established methods (Zeng et al., 2007). Two hundred mature embryo sacs were selected from each line (H1 and *ntrf1* mutants) for observation using WE‐CLSM (Leica SPE, Leica Microsystems, Germany). Mature pollen grains were stained with 1% I_2_‐KI and 1% TTC solutions, then observed and imaged using a Motic BA210 microscope. Pollen fertility assessment was performed on 15 independent rice plants. For semi‐thin section analysis, following dehydration through an ethanol series, tissue specimens were embedded using the Technovit 7100 embedding kit (Heraeus‐Kulzer, Heraeus, Germany) according to the manufacturer's instructions. Sections with a thickness of 3 to 4 μm were cut using a microtome (Leica RM2235, Leica Microsystems, Germany) and dried at 60°C for 24 h. The sections were then stained with 0.5% toluidine blue. Stained sections were observed and photographed using an optical microscope (Motic BA210, Motic China Group, China).

For TEM observation, meiotic and mature anthers were placed in pre‐cooled glutaraldehyde fixative overnight, then rinsed four times with 0.1 M phosphate buffer. The samples were subsequently fixed with 1% osmic acid for 2 h, followed by rinsing four times with double‐distilled water. The blocks were stained with 0.25% to 1% uranyl acetate at 4°C overnight. The anther samples were dehydrated using a gradient ethanol solution, followed by sequential immersion in the embedding medium (acetone: resin = 3:1 for 4 h; 2:1 for 4 h; 1:1 for 8 h; 0:1 for 24 h, v/v). The embedding medium was polymerized in a 70°C oven for 24 h. Ultrathin sections and double staining were conducted at the Instrument Testing Center of South China Agricultural University. Finally, the samples were observed under a transmission electron microscope (Talos L120C, Thermo Fisher Scientific, Czech).

### TUNEL assay

The TUNEL assay was conducted to detect DNA fragmentation. Anthers at various developmental stages were fixed in 4% paraformaldehyde (in PBS). The fixed samples were then embedded, and sections were prepared using a cryostat. The slide sections were washed twice with PBS for 15 min per wash. Subsequently, a 100 μL solution of Proteinase K at a concentration of 20 μg/mL was added dropwise to each sample for incubation. DNA strand breaks were detected using the TUNEL Apoptosis Detection Kit (YSFluor^TM^ 488, Yeasen, China) following the manufacturer's instructions. Finally, the stained sections were observed under a laser‐scanning microscope (Leica DM 2500, Leica Microsystems, Germany). All photographs were taken with identical settings.

### RT‐qPCR analysis

Total RNA was extracted using TRIzol reagent (AG, China), and first‐strand cDNA was synthesized with a reverse transcription kit (Hifair® III 1st Strand cDNA Synthesis SuperMix for qPCR, Yeasen, China). RT‐qPCR was performed using the Hieff qPCR SYBR Green Master Mix (Yeasen, China) and the Light‐Cycler 480II system (Roche, Switzerland) in accordance with the manufacturer's instructions. Relative expression levels were calculated using the 2^−ΔΔCt^ method (Livak and Schmittgen, 2001), with the rice ubiquitin gene as the internal control. Three biological replicates and three technical replicates were included for each sample. The sequences of primers used for RT‐qPCR are listed in Table S2.

### Transcriptome analysis

Samples of H1 and *ntrf1* mutants were collected from S7 anthers (glume length of 3.5–4.3 mm), S8 anthers (glume length of 4.5–5.5 mm), S9 anthers (glume length of 6–7 mm), and S11 anthers (glume length of 7.5–8 mm). Anther developmental stages were classified based on well‐established morphological criteria (Zhang et al., 2011). Each sample included three biological replicates and was stored at −80°C. Total RNA was extracted using established methods (Chen et al., 2019) for subsequent library construction. The quality of sequencing libraries was evaluated using the Agilent Bioanalyzer 2100 system (Agilent Technologies, USA), and sequencing was performed on the Illumina NovaSeq 6000 platform (San Diego, CA, USA). The data were processed using the bioinformatics analysis platform BMK‐Cloud (Beijing, China) and aligned to the Nipponbare genome (MSU7.0). DEGs were identified based on |Log_2_FC | ≥ 1 and a significant difference (*P* < 0.01). Heatmaps were generated using TBtools‐II software (Chen et al., 2023a).

### Metabolome analysis

Mature anther samples from both H1 and *ntrf1* mutants were collected and stored on dry ice. Following quality inspection, metabolites were identified using mass spectrometry. The raw mass spectrometry data files were converted to mzXML format using the MSConvert tool from the Proteowizard software package (version 3.0.8789) (Rasmussen et al., 2022). The R XCMS software (version 3.12.0) package was utilized for data processing to generate the list of quantified substances (Navarro‐Reig et al., 2015). Substances were identified in the database with the parameter ppm < 30. Differential screening of primary and secondary metabolites was conducted with thresholds of *P* < 0.05 and variable importance for the projection (VIP) > 1. Heatmaps of differential metabolites and pathway analysis diagrams were generated using the Chengqi Medical Analysis Platform (Shenzhen, China).

### ROS levels analysis

To assess superoxide anion accumulation, anther samples at distinct developmental stages were collected from H1 and *ntrf1‐1* mutants. The anthers were immersed in NBT solution (Coolaber, SL1806, China), subjected to vacuum infiltration for 5 min, and subsequently incubated at 25°C for 6 h in the dark. Additional staining was performed using H_2_DCF‐DA (MedChemExpress, 4091‐99‐0, USA). Tissues were immersed in 5 μM H_2_DCF‐DA working solution, vacuum‐infiltrated, and incubated for 1 h at room temperature in the dark. The staining solution was then removed, and the samples were immediately visualized under a fluorescence stereomicroscope (Nikon Tripolls 100, Nikon Corporation, Japan). Quantitative analysis of fluorescence intensity in H_2_DCF‐DA‐stained anthers was conducted using ImageJ software to reflect ROS levels. Approximately 90 anthers per developmental stage were examined.

### Subcellular localization

Subcellular localization analysis was performed as described in the previous research (Yoo et al., 2007). The recombinant vectors NTRF1‐pAN580 and SAPK2‐pAN580 were transiently transformed into rice protoplasts using the polyethylene glycol‐mediated method, with SPER‐mKATE used as a marker. After approximately 18 h of incubation in darkness, the GFP fluorescence was observed using a laser scanning confocal microscope (Nikon C2‐ER, Nikon Corporation, Japan).

### BiFC assay

The CDS of *OsNTRF1* (LOC_Os05g44340) and *OsSAPK2* (LOC_Os07g42940) were cloned into the pGreenII‐62‐SK‐VC and pGreenII‐62‐SK‐VN vectors, respectively, and fused to generate the recombinant constructs NTRF1‐cYFP and SAPK2‐nYFP. The recombinant vectors were transformed into rice protoplasts. After approximately 18 h of incubation in darkness, the fluorescence signals were observed under a laser scanning confocal microscope (Nikon C2‐ER, Nikon Corporation, Japan).

### LCI assay

The CDS sequences of *OsNTRF1* and *OsSAPK2* were inserted into pCAMBIA1300‐nLUC and pCAMBIA1300‐cLUC vectors to construct NTRF1‐nLUC and SAPK2‐cLUC recombinant vectors. These constructs were transformed into the *Agrobacterium tumefaciens* strain (GV3101) and then co‐infiltrated into *Nicotiana benthamiana* leaves. The leaves were removed after 3 days of infestation and sprayed with a working solution (1 mM D‐Luciferin potassium salt, Promega, P1043, USA). The leaves were observed using a CCD imaging instrument (Berthold, NightSHADE LB985, Germany).

### Co‐IP assay

The CDS of *OsNTRF1* and *OsSAPK2* were cloned into the pCAMBIA1300‐3×Flag and pCAMBIA1302 vectors, respectively. The recombinant plasmids were transformed into the competent *Agrobacterium tumefaciens* strain GV3101. Resuspended bacterial suspensions were mixed in equal volumes and infiltrated into leaves of *Nicotiana benthamiana* plants. Leaves were harvested after two days, ground in liquid nitrogen, and homogenized in NB1 buffer (50 mM Tris‐MES, pH 8.0, 500 mM sucrose, 1 mM MgCl_2_, 10 mM EDTA, 5 mM DTT, 1 mM PMSF, 100× Cocktail). The homogenate was centrifuged to yield the crude protein extract. The NB1 extract was incubated with anti‐Flag magnetic beads for 3 h at 4°C with rotation. The beads were washed three times with NB1 buffer supplemented with protease inhibitors. A volume of 5× protein loading buffer was added to the beads at a 1:2 ratio, followed by boiling at 100°C for 5 min. Proteins were separated by SDS‐PAGE and subsequently analyzed by western blot. Immunodetection was performed using anti‐Flag antibody and anti‐HA antibody. Finally, signals were detected and imaged using a CCD imaging system (Berthold NightSHADE LB985, Germany).

### Primers

The relevant primers used are listed in Table S2.

### Generative AI usage statement

During the preparation of this work, the authors utilized the DeepSeek AI assistant for the purpose of improving the language. After using this tool, the authors carefully reviewed and revised the content where necessary and took full responsibility for the final version of the publication.

## CONFLICTS OF INTEREST

The authors declare no conflicts of interest.

## AUTHOR CONTRIBUTIONS

L.C., W.H., S.L., and J.Y. performed the experiments. L.C., W.H., and Z.L. analyzed the data. H.Y. and J.W. contributed to the methodology. X.L. developed NTR. L.C. and X.L. wrote the manuscript. X.L. and L.C. conceived and designed the experiments. All authors have read and approved the final version of the manuscript.

## Supporting information

Additional Supporting Information may be found online in the supporting information tab for this article: http://onlinelibrary.wiley.com/doi/10.1111/jipb.70218/suppinfo

**Figure S1.** Targeted mutagenesis of *NTRF1* and the schematic diagram of its complementation line
**Figure S2.** The SNP variations in the *NTRF1* gene lead to three distinct changes in neo‐tetraploid rice (NTR) compared to *HSP101* in diploid rice
**Figure S3.** Phenotypic characterization of *hsp101* in diploid rice
**Figure S4.** Scanning electron microscopy (SEM) analysis of pollen grains from Huaduo1 (H1) and *ntrf1*
**Figure S5.** Analysis of programmed cell death (PCD) in anthers of Huaduo1 (H1) and *ntrf1*
**Figure S6.** RT‐qPCR analysis of ABA‐related differentially expressed genes (DEGs)
**Figure S7.** RT‐qPCR analysis of ROS‐related differentially expressed genes (DEGs)
**Figure S8.** Kyoto Encyclopedia of Genes and Genomes (KEGG) pathway analysis of downregulated DEGs in anthers
**Figure S9.** RT‐qPCR analysis of pollen development‐related differentially expressed genes (DEGs)
**Figure S10.** Metabolomic analysis of the anthers at the S12 stage
**Figure S11.** Yeast two‐hybrid analysis of NTRF1 and SAPK2
**Figure S12.** Endogenous abscisic acid (ABA) quantification was conducted via UPLC‐MS/MS
**Table S1.** mRNA‐seq sample quality information
**Table S2.** Primers used in this study

## Data Availability

The raw reads of RNA‐seq were deposited in the NGDC BIG Submission with accession ID PRJCA058658. The sequences and annotations of the rice *japonica* reference genome MSU7 are available from the website: http://rice.plantbiology.msu.edu/.
