# Supplementary material for: HSP101‐encoding NEO‐TETRAPLOID RICE FERTILITY GENE 1 regulates tapetum development through interaction with SAPK2 in polyploid rice
Source: J Integr Plant Biol. 2026 Mar 11;68(7):2316–30. doi: 10.1111/jipb.70218 (PMC13326973; doi:10.1111/jipb.70218)
Supplement: Supplementary file 1 — Figure S1. Targeted mutagenesis of NTRF1 and the schematic diagram of its complementation line Figure S2. The SNP variations in the NTRF1 gene lead to three distinct changes in neo‐tetraploid rice (NTR) compared to HSP101 in diploid rice Figure S3. Phenotypic characterization of hsp101 in diploid rice Figure S4. Scanning electron microscopy (SEM) analysis of pollen grains from Huaduo1 (H1) and ntrf1 Figure S5. Analysis of programmed cell death (PCD) in anthers of Huaduo1 (H1) and ntrf1 Figure S6. RT‐qPCR analysis of ABA‐related differentially expressed genes (DEGs) Figure S7. RT‐qPCR analysis of ROS‐related differentially expressed genes (DEGs) Figure S8. Kyoto Encyclopedia of Genes and Genomes (KEGG) pathway analysis of downregulated DEGs in anthers Figure S9. RT‐qPCR analysis of pollen development‐related differentially expressed genes (DEGs) Figure S10. Metabolomic analysis of the anthers at the S12 stage Figure S11. Yeast two‐hybrid analysis of NTRF1 and SAPK2 Figure S12. Endogenous abscisic acid (ABA) quantification was conducted via UPLC‐MS/MS Table S1. mRNA‐seq sample quality information Table S2. Primers used in this study [file JIPB-68-2316-s001.docx]

***HSP101*-encoding NEO-TETRAPLOID RICE FERTILITY GENE 1 regulates tapetum development through interaction with SAPK2 in** **polyploid rice**

Lichong Cao^1,2,3†^, Weicong Huang^1,2,3†^, Hang Yu^1,2,3^, Sanglin Liu^1,2,3^, Jianmin Yin^1,2,3^, Zijun Lu^1,2,3^, Jinwen Wu^1,2,3^, Xiangdong Liu^1,2,3*^

**SUPPORTING INFORMATION**


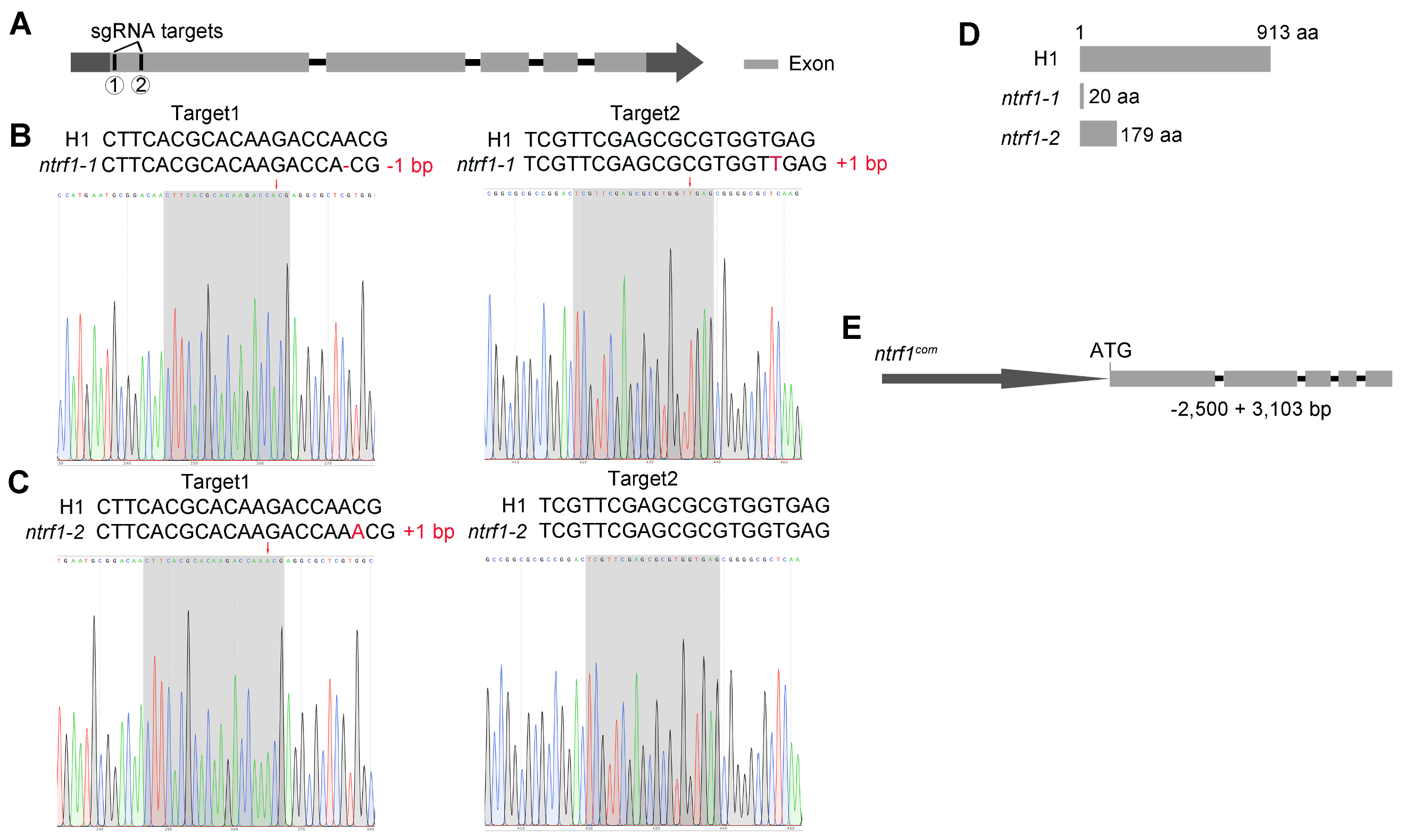


**Figure S1. Targeted mutagenesis of *NTRF1* and the schematic diagram of its complementation line**

**(A)** Schematic diagram of *NTRF1* gene editing. Two sgRNAs targeting *NTRF1* were designed, Target site positions are indicated on the gene structure. **(B, C)** Two mutants, *ntrf1‐1* and *ntrf1‐2*, were generated using the clustered regularly interspaced short palindromic repeats (CRISPR)/CRISPR-associated protein 9 (Cas9) system. The red arrows indicate the position of the base deletion or insertion. **(D)** Schematic diagram of the amino acid sequences of the *ntrf1-1* and *ntrf1-2* mutants. **(E)** The 5,603 bp genomic DNA fragment of *NTRF1* was introduced into the *ntrf1-1* mutant to construct the complementation transgenic line (*ntrf1^com^*).


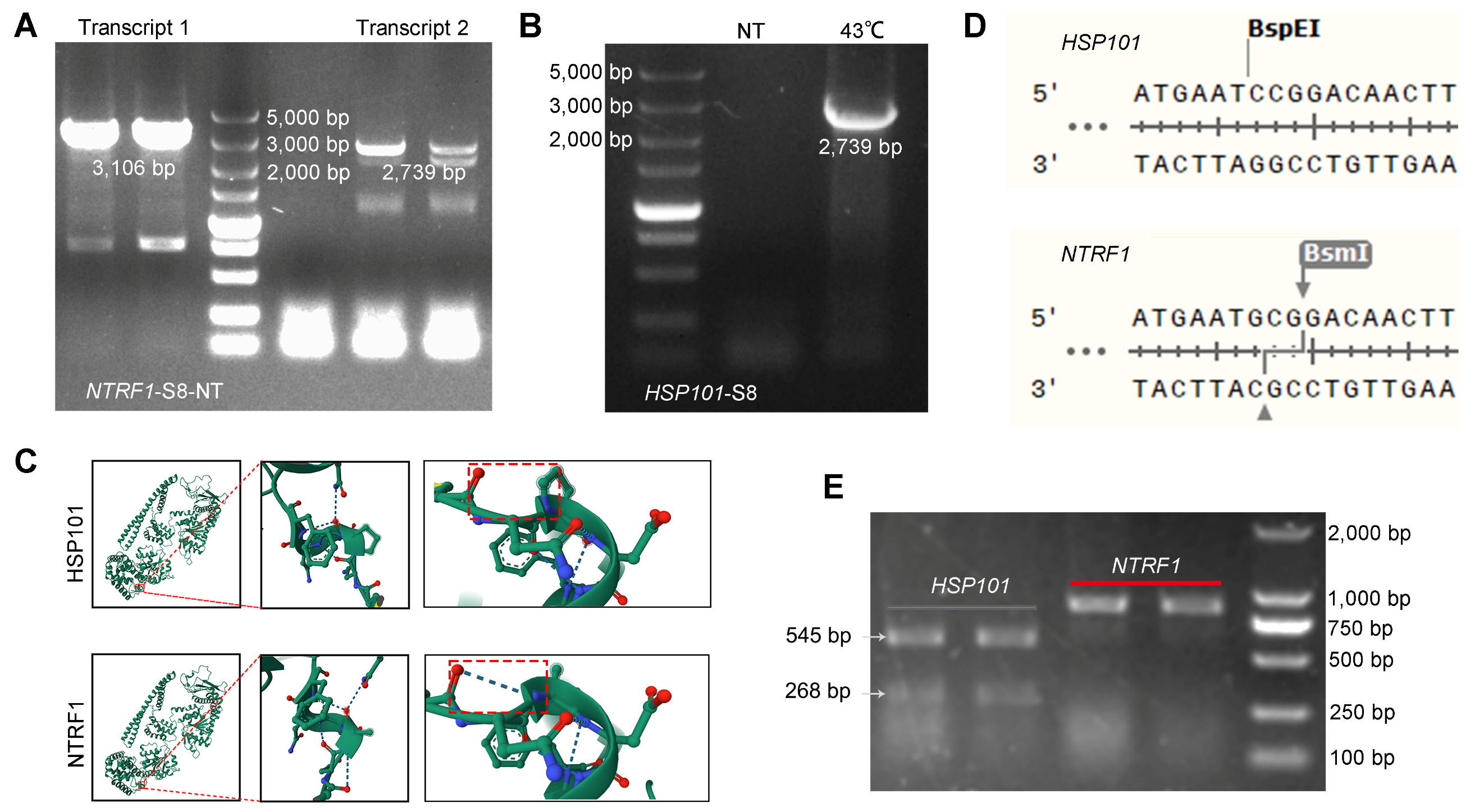


**Figure S2. The SNP variations in the *NTRF1* gene lead to three distinct changes in neo-tetraploid rice (NTR) compare to *HSP101* in diploid rice**

**(A)** The coding sequences (CDS) amplification of *NTRF1* revealed two transcripts, one fully spliced transcript with a length of 2,739 bp, and another transcript retaining all introns, with a length of 3,106 bp. S8, Stages of anther development; NT, normal temperature. **(B)** The CDS amplification of *HSP101* in diploid rice showed that in diploid rice could only detect 2,739 bp transcripts after heat shock treatment. **(C)** The tertiary structures of HSP101 and NTRF1 were predicted using AlphaFold2. **(D)** Single nucleotide polymorphism (SNP) variation leads to changes in restriction enzyme cleavage sites. **(E)** Polymerase chain reaction (PCR) was used to amplify the target band containing SNP loci with a total length of 813 bp. BspEI restriction endonuclease was used to cleave the target fragment, showing that *HSP101* could be cleaved into two bands of 268 bp and 545 bp, respectively. *NTRF1* only displayed one band of 813 bp in length.


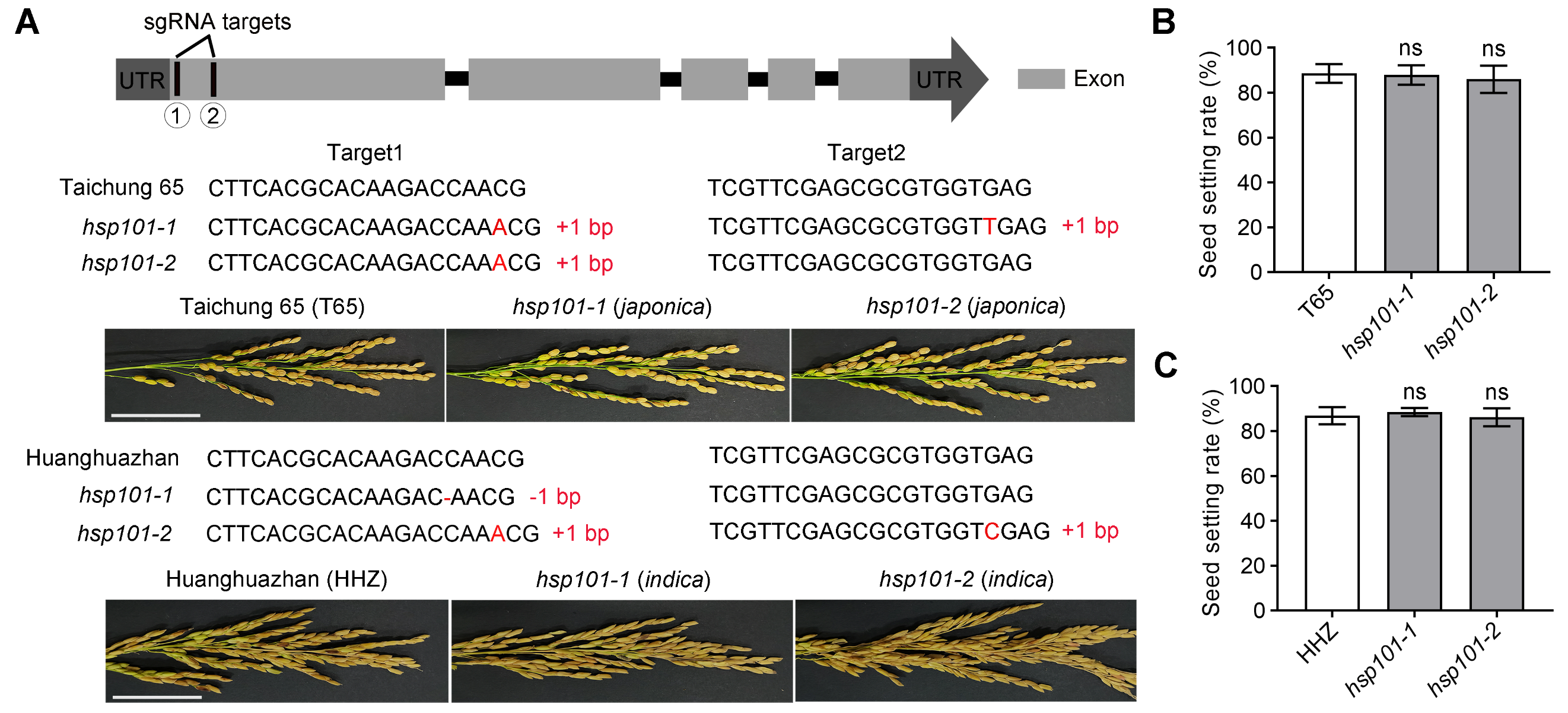


**Figure S3. Phenotypic characterization of *hsp101* in diploid rice**

**(A)** Schematic diagram of sequence variation and mature panicles of the *hsp101* mutant under two diploid rice backgrounds (Taichung 65 and Huanghuazhan). Bars = 5 cm. **(B)** Statistical results of seed-setting rate in T65 and *hsp101* mutants (means ± *SD*, **P* < 0.05, ***P* < 0.01, two-tailed Student's *t*-test, *n* = 15). **(C)** Statistical results of seed-setting rate in HHZ and *hsp101* mutants (means ± *SD*, **P* < 0.05, ***P* < 0.01, two-tailed Student's *t*-test, *n* = 15).

**
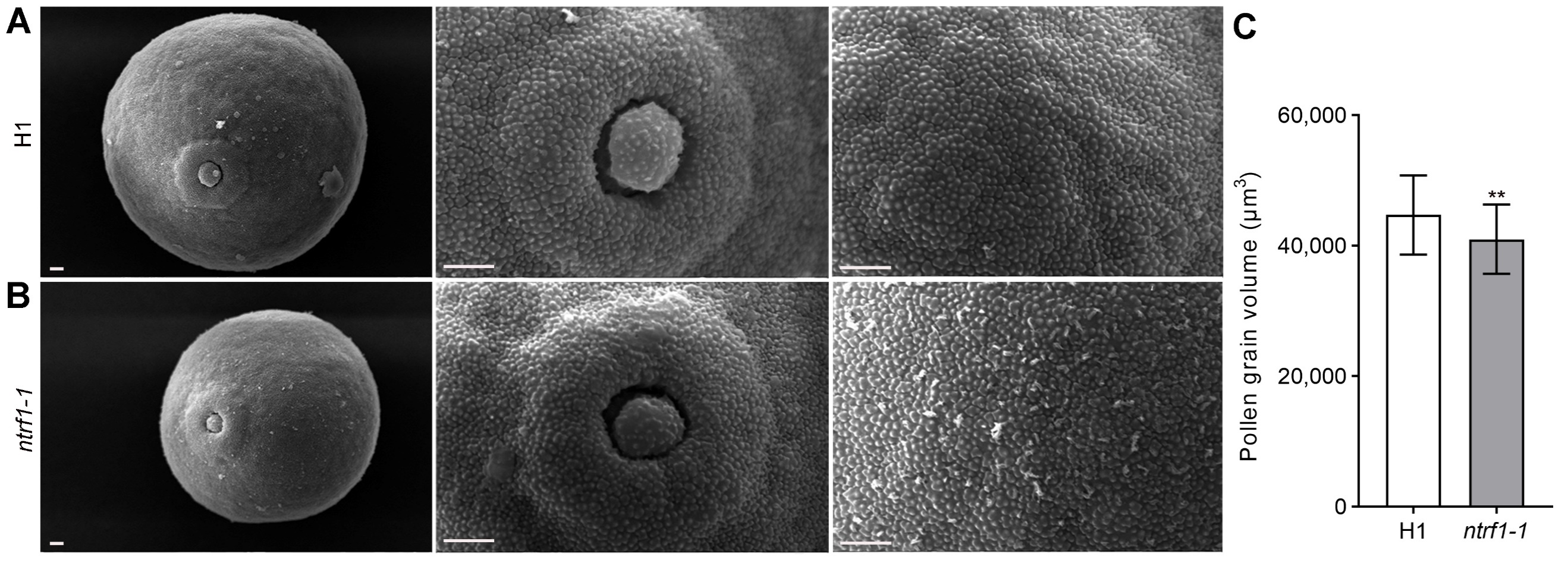
**

**Figure S4.** **Scanning electron microscopy (SEM) analysis of pollen grains from Huaduo 1 (H1) and *ntrf1***
**(A, B)** Morphology of mature pollen grains from H1 and *ntrf1-1* mutant observed using scanning electron microscopy (SEM). Bars = 2 μm. **(C)** Pollen grain volume in H1 and the *ntrf1-1* mutant (means ± *SD*, **P* < 0.05, ***P* < 0.01; two-tailed Student's *t*-test, *n* = 95).


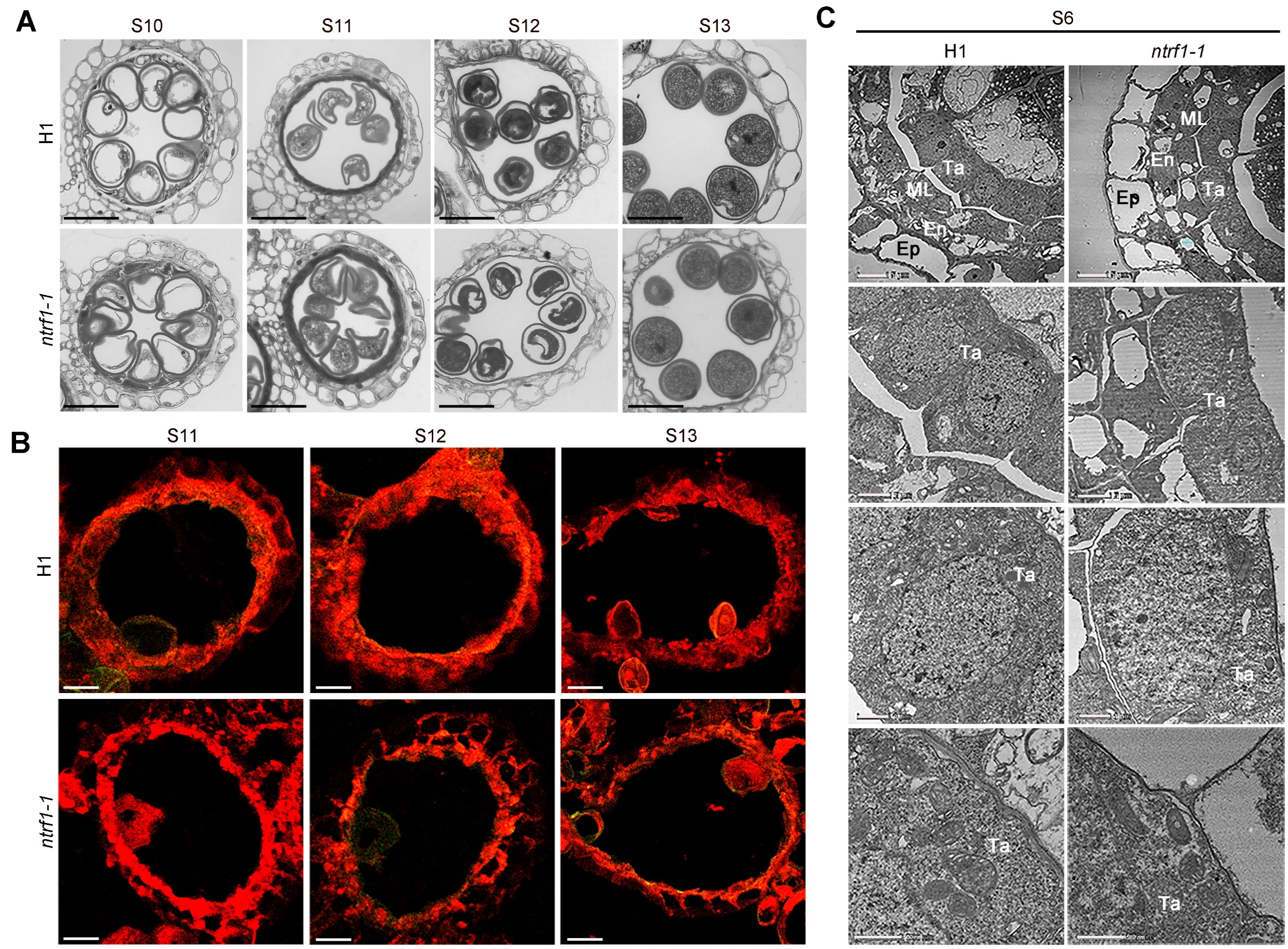


**Figure S5. Analysis of programmed cell death (PCD) in anthers of Huaduo 1 (H1) and *ntrf1***

**(A)** Semi-thin section analysis of anther development at the S10 to S13 stages in H1 and *ntrf1*, respectively. Bars = 50 μm. **(B)** Terminal deoxynucleotidyl transferase-mediated dUTP nick-end labeling (TUNEL) staining characteristics of anther development at S11-S13 in H1 and *ntrf1*, respectively. Bars = 100 μm. **(C)** Transmission electron microscopy (TEM) analysis of anther wall from H1 and *ntrf1-1* mutant at S6. The scale bars in the figure, from top to bottom, are 5 μm, 2 μm, 1 μm, and 500 nm, respectively. They also appear in the lower left corner of each image. Ep, epidermis; En, endothecium; ML, middle layer; Ta, tapetum.


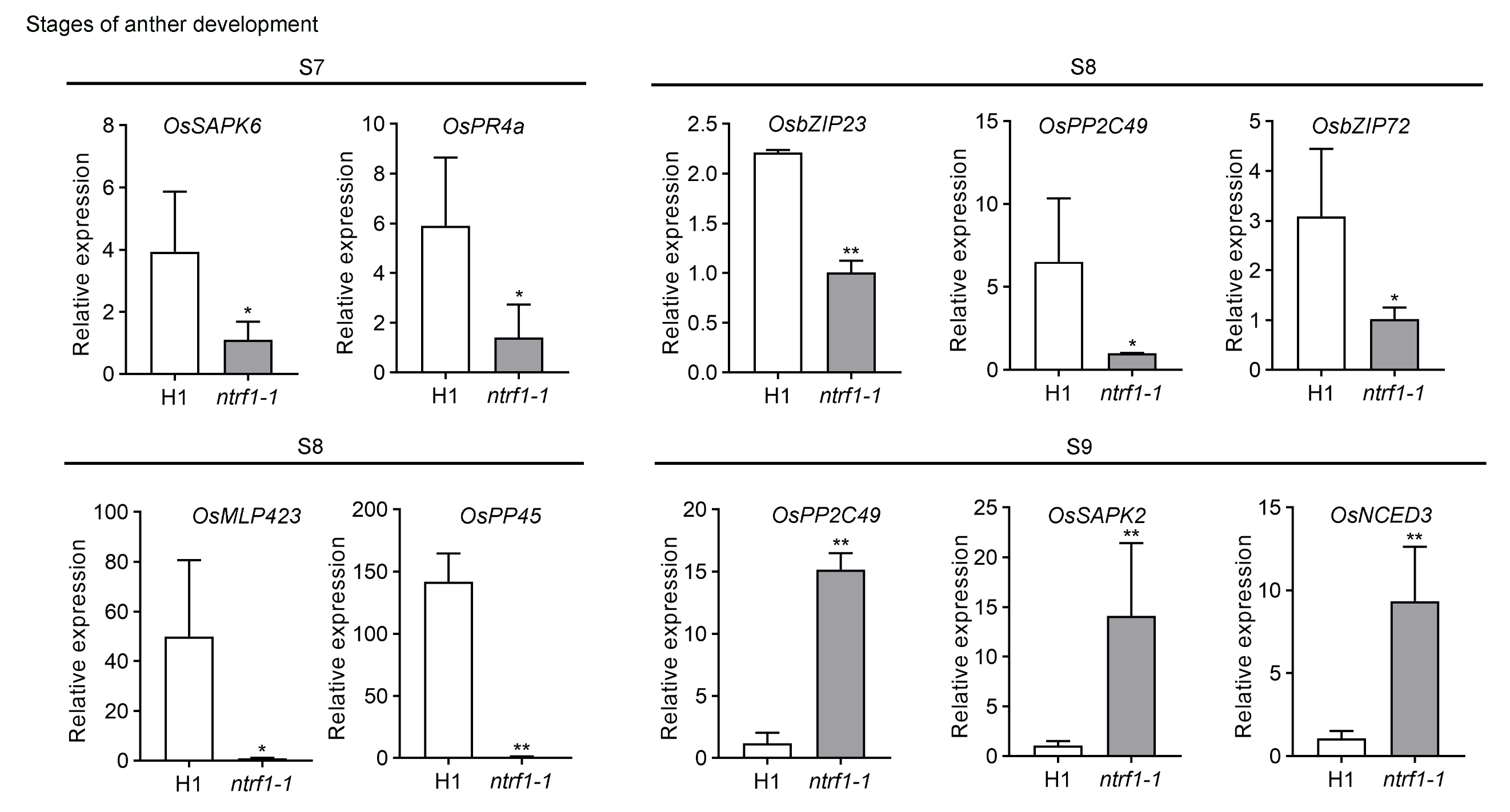


**Figure S6.** **RT-qPCR analysis of ABA-related differentially expressed genes (DEGs)**
Relative expression levels of ABA-related genes in H1 and *ntrf1-1* mutant during the S7 to S9 stages of pollen development (means ± *SD*, **P* < 0.05, ***P* < 0.01, two‐tailed Student's *t*‐test, *n* = 3).


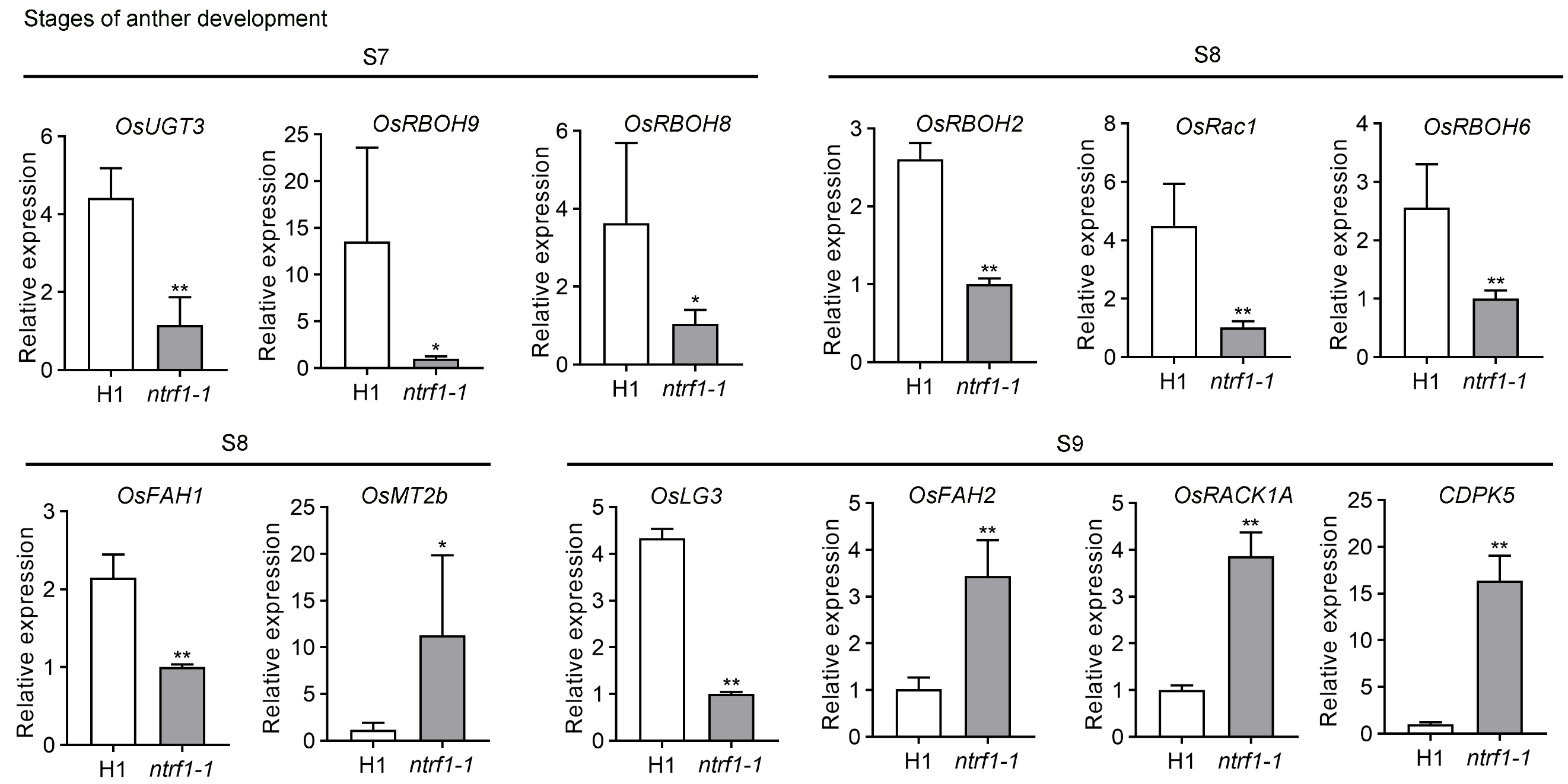


**Figure S7. RT-qPCR analysis of ROS-related differentially expressed genes (DEGs)**

Relative expression levels of ROS-related genes in H1 and *ntrf1-1* mutant during the S7 to S9 stages of pollen development (means ± *SD*, **P* < 0.05, ***P* < 0.01, two‐tailed Student's *t*‐test, *n* = 3).


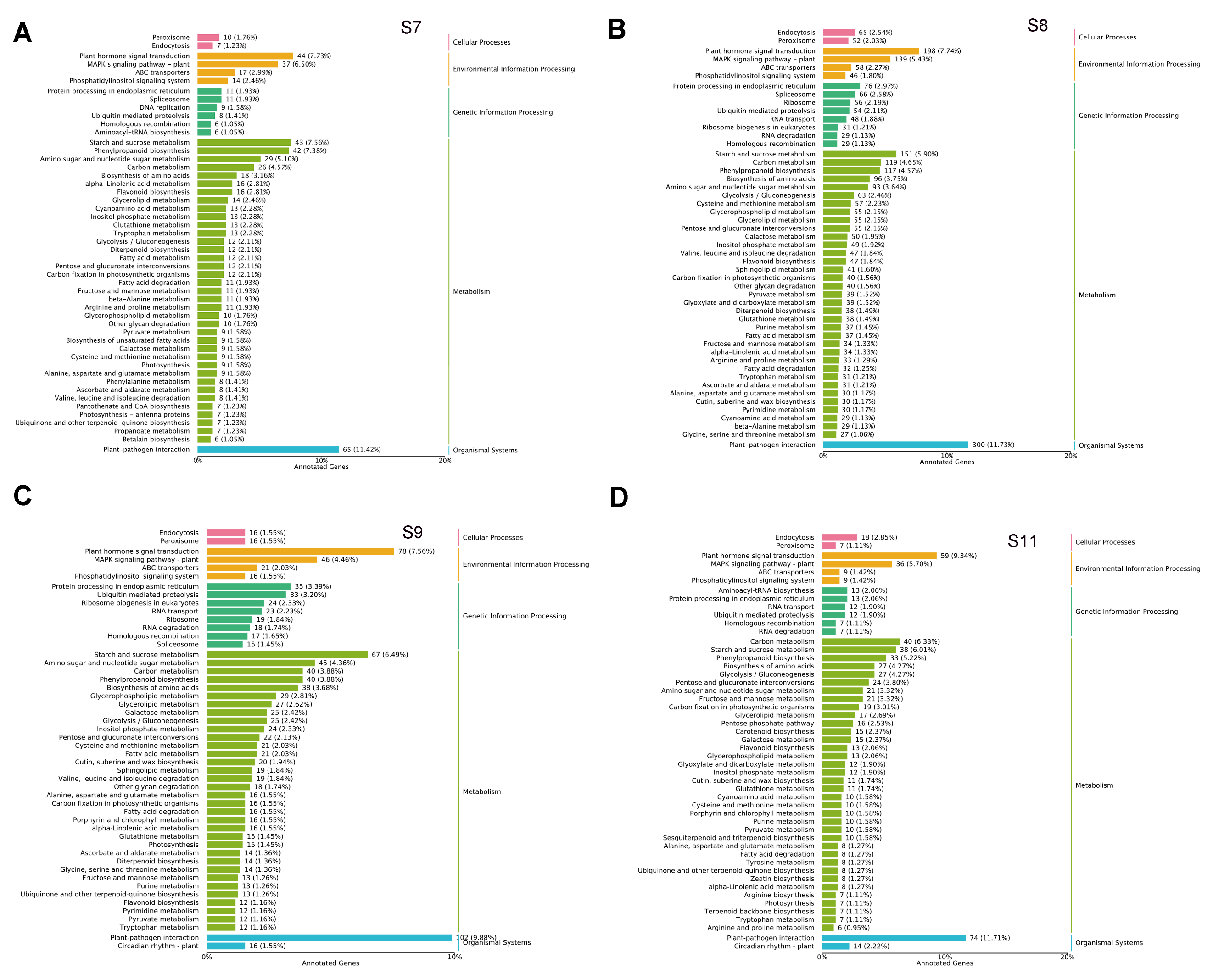


**Figure S8. Kyoto Encyclopedia of Genes and Genomes (KEGG) pathway analysis of downregulated DEGs in anthers
(A-D)** KEGG pathway analysis of downregulated DEGs in H1 and *ntrf1-1* mutant during the S7 stage **(A)**, S8 stage **(B)**, S9 stage **(C)**, and S11 stage **(D)**, respectively.


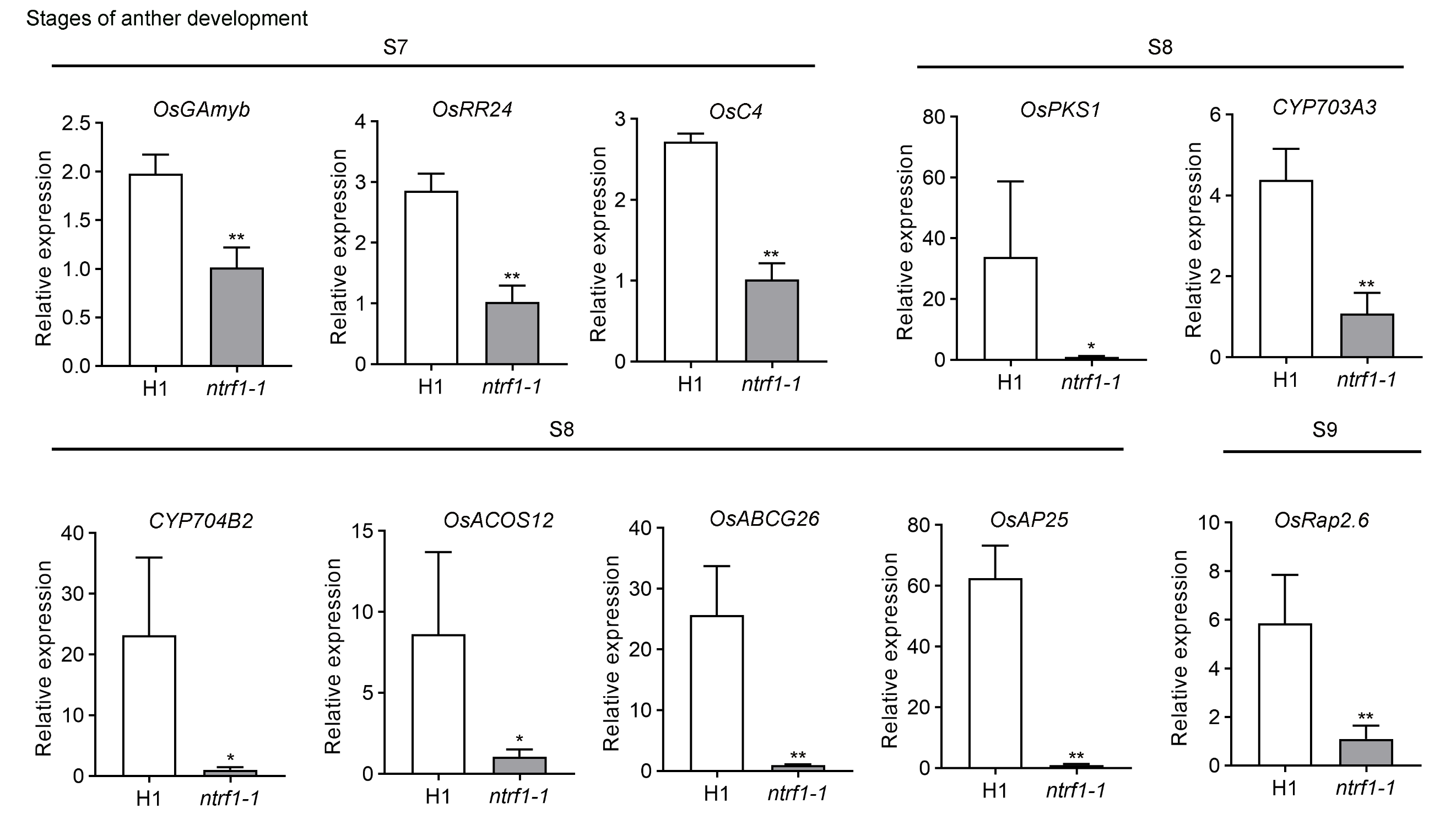


**Figure S9. RT-qPCR analysis of pollen development-related differentially expressed genes (DEGs)**

Relative expression levels of pollen development-related genes in H1 and *ntrf1-1* mutant during stages S7 to S9 (means ± *SD*, **P* < 0.05, ***P* < 0.01, two‐tailed Student's *t*‐test, *n* = 3).


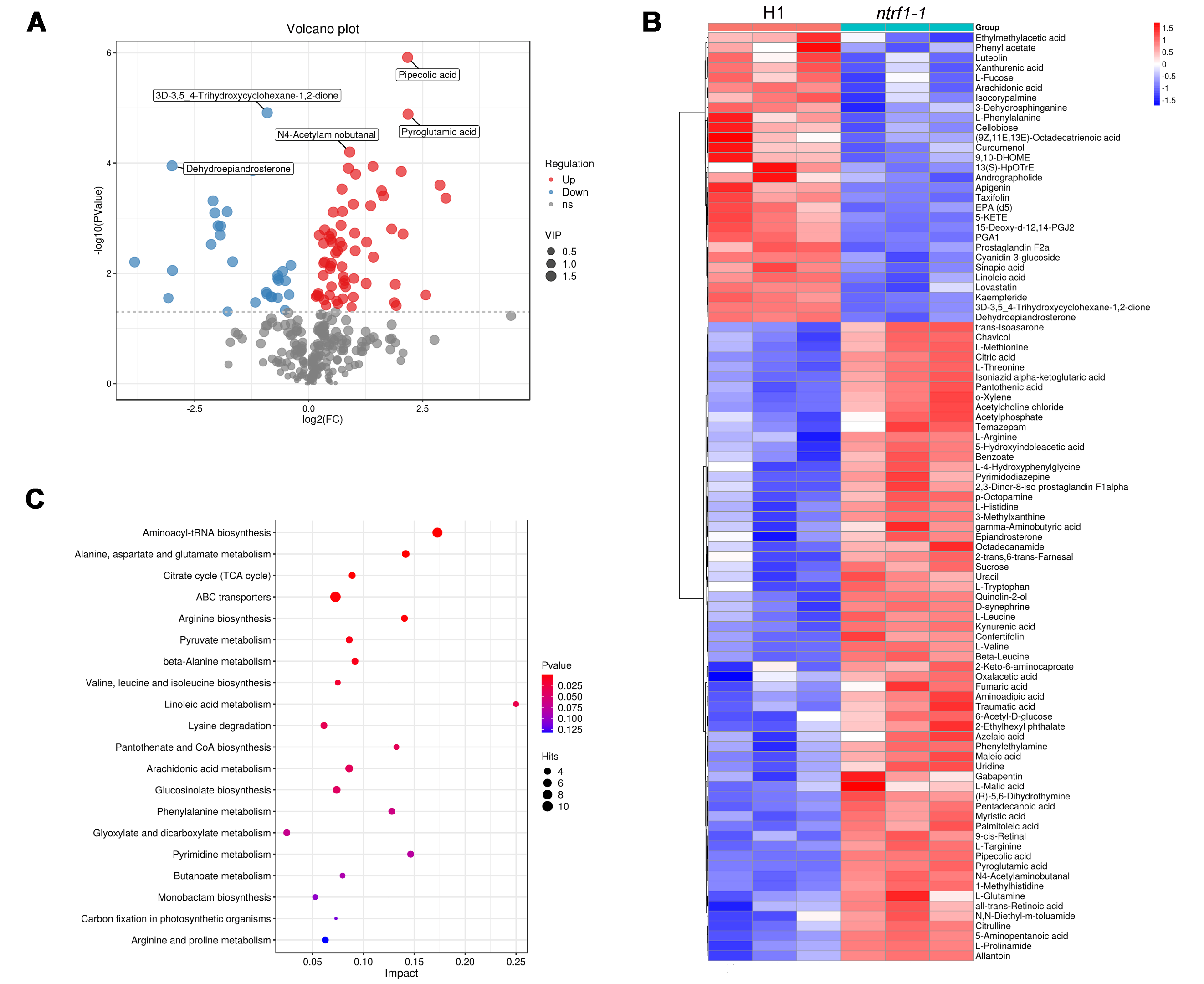


**Figure S10. Metabolomic analysis of the anthers at the S12 stage**
**(A)** Volcano plot of differentially accumulated metabolites between H1 and *ntrf1* mutant. **(B)** Hierarchical clustering heatmap of differentially accumulated metabolites. The gradient color represents the magnitude of the quantitative values. **(C)** Kyoto Encyclopedia of Genes and Genomes (KEGG) pathway analysis of differentially accumulated metabolites.


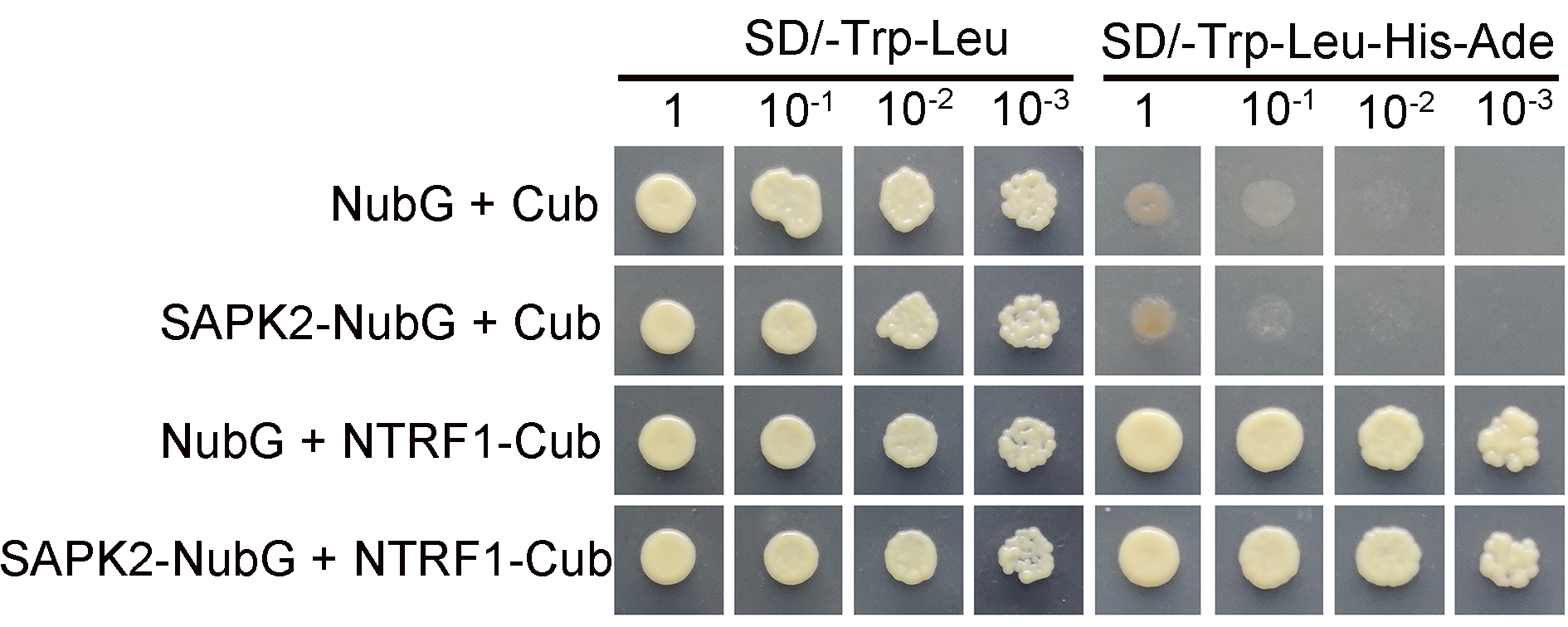


**Figure** **S11. Yeast** **two-hybrid analysis of NTRF1 and SAPK2**
The yeast two-hybrid assay showed the interaction between NTRF1 and SAPK2, demonstrating that NTRF1 exhibits auto-activation activity in the membrane yeast two-hybrid system.

**
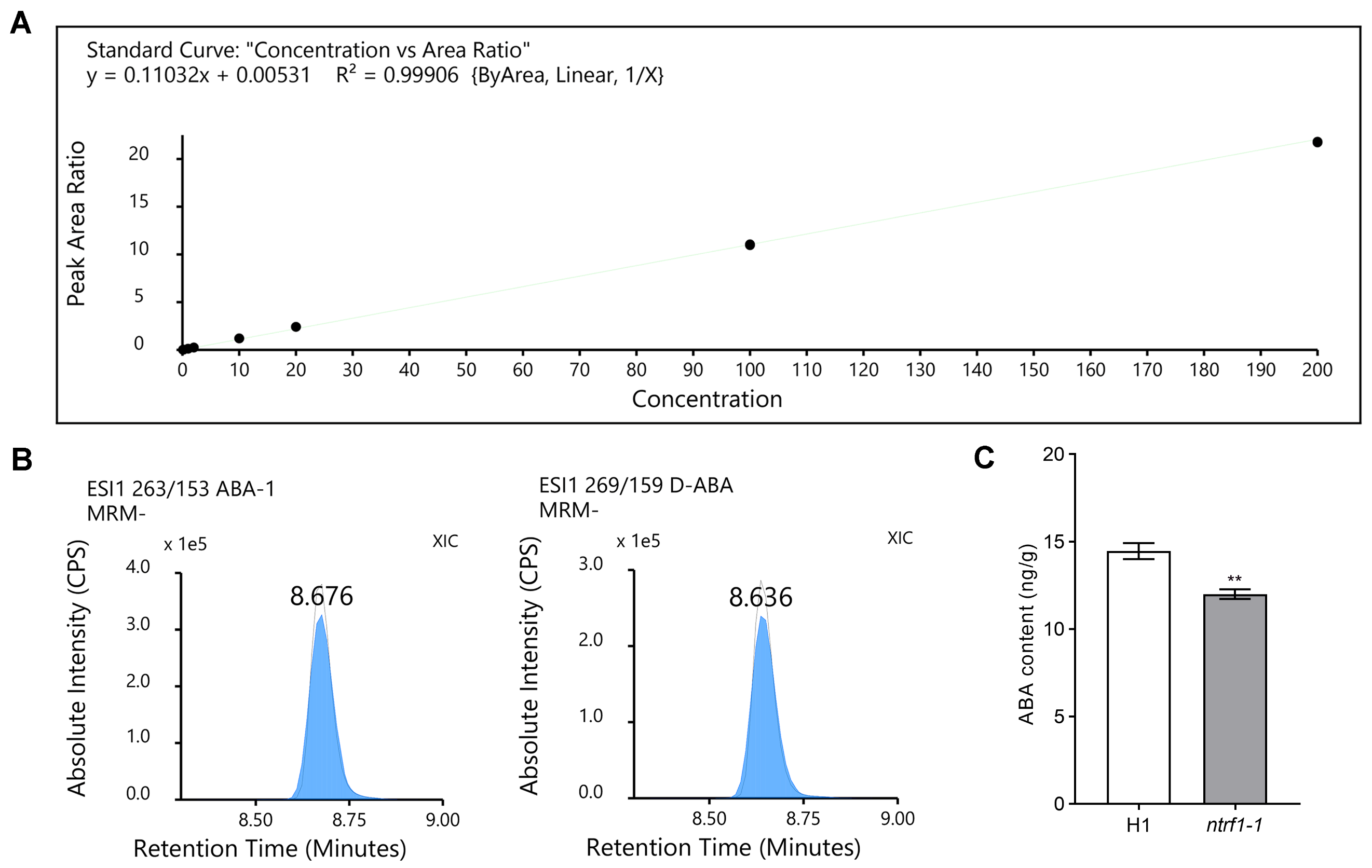
**

**Figure S12. Endogenous abscisic acid (ABA) quantification was conducted via UPLC-MS/MS**

**(A)** Calibration curves (*R*² = 0.999) were generated by correlating analyte-to-internal standard peak area ratios of characteristic ion transitions with authentic standard concentrations, establishing the vertical axis as peak area ratio and the horizontal axis as concentration. **(B)** Representative extracted ion chromatograms (XIC) for target analytes and internal standards are shown. **(C)** Comparative analysis revealed that endogenous ABA levels in meiotic anthers were significantly lower in the *ntrf1-1* mutant compared to the H1 (means ± *SD*, ***P* < 0.01, two‐tailed Student's *t*‐test, *n* = 3).

**Table S1.** **mRNA-seq sample quality information**

| **Samples** | **Clean reads** | **Clean bases** | **%≥Q30** | **Total Reads** | **Mapped Reads** |
| --- | --- | --- | --- | --- | --- |
| H1-S7-1 | 24,718,472 | 7,400,004,870 | 93.41% | 49,436,944 | 95.38% |
| H1-S7-2 | 27,963,109 | 8,372,106,142 | 93.79% | 55,926,218 | 95.64% |
| H1-S7-3 | 24,526,288 | 7,342,817,952 | 93.77% | 49,052,576 | 95.62% |
| *ntrf1*-S7-1 | 23,986,988 | 7,181,777,968 | 93.71% | 47,973,976 | 95.61% |
| *ntrf1*-S7-2 | 20,575,318 | 6,158,991,598 | 93.99% | 41,150,636 | 95.33% |
| *ntrf1*-S7-3 | 21,951,413 | 6,572,594,980 | 93.80% | 43,902,826 | 95.77% |
| H1-S8-1 | 22,657,216 | 6,775,189,974 | 92.77% | 45,314,432 | 94.48% |
| H1-S8-2 | 20,583,987 | 6,150,267,694 | 92.64% | 41,167,974 | 94.71% |
| H1-S8-3 | 21,162,132 | 6,324,183,422 | 93.16% | 42,324,264 | 94.86% |
| *ntrf1*-S8-1 | 21,471,179 | 6,415,021,662 | 92.47% | 42,942,358 | 93.84% |
| *ntrf1*-S8-2 | 19,682,798 | 5,881,016,882 | 92.44% | 39,365,596 | 94.25% |
| *ntrf1*-S8-3 | 22,274,378 | 6,654,089,600 | 92.43% | 44,548,756 | 93.54% |
| H1-S9-1 | 20,788,022 | 6,224,353,136 | 93.97% | 41,576,044 | 95.52% |
| H1-S9-2 | 25,783,210 | 7,719,237,918 | 93.64% | 51,566,420 | 94.96% |
| H1-S9-3 | 24,748,476 | 7,407,597,636 | 93.78% | 49,496,952 | 95.20% |
| *ntrf1*-S9-1 | 23,144,946 | 6,930,992,210 | 93.73% | 46,289,892 | 95.73% |
| *ntrf1*-S9-2 | 26,486,398 | 7,930,646,168 | 93.55% | 52,972,796 | 95.54% |
| *ntrf1*-S9-3 | 22,985,711 | 6,881,328,266 | 93.61% | 45,971,422 | 95.79% |
| H1-S11-1 | 29,370,807 | 8,796,658,720 | 95.49% | 58,741,614 | 94.71% |
| H1-S11-2 | 26,853,011 | 8,045,049,615 | 95.28% | 53,706,022 | 94.88% |
| H1-S11-3 | 23,144,802 | 6,931,826,203 | 95.44% | 46,289,604 | 95.20% |
| *ntrf1*-S11-1 | 22,541,436 | 6,750,937,726 | 95.55% | 45,082,872 | 94.89% |
| *ntrf1*-S11-2 | 29,439,110 | 8,819,079,500 | 95.27% | 58,878,220 | 94.87% |
| *ntrf1*-S11-3 | 25,760,126 | 7,716,105,370 | 94.95% | 51,520,252 | 94.30% |

| **Primer name** | **Primer sequence (5’-3’)** |
| --- | --- |
| *NTRF1*-T1-F | CTCGCCTCTTCTCGTCCTTC |
| *NTRF1*-T2-R | CTCACCACGCGCTCGAACGA |
| *NTRF1*-GFP-F | ggacagcccagatcaactagtATGAATGCGGACAACTTCACG |
| *NTRF1*-GFP-R | gcccttgctcaccatggatccCTCTTCGTCCATGCCGTCC |
| *SAPK2*-GFP-F | ggacagcccagatcaactagtATGGAGAGGTACGAGGTGATCAA |
| *SAPK2*-GFP-R | gcccttgctcaccatggatccCAATGCGCACACGAAGTCG |
| *SAPK2*-NubG-F | gccatggaggccagtgaattcATGGAGAGGTACGAGGTGATCAA |
| *SAPK2*-NubG-R | cagctcgagctcgatggatccTCACAATGCGCACACGAAGT |
| *NTRF1*-Cub-F | atatggccatggaggccATGAATGCGGACAACTTCACGCACAAGACC |
| *NTRF1*-Cub-R | ccgctgcaggtcgacCTACTCTTCGTCCATGCCGTCCTCG |
| *NTRF1*-P2YC-F  *NTRF1*-P2YC-R  *SAPK2*-P2YN-F  *SAPK2*-P2YN-R  nLUC-*NTRF1*-F  nLUC-*NTRF1*-R  cLUC-*SAPK2*-F  cLUC-*SAPK2*-R  *NTRF1*-Flag-F  *NTRF1*-Flag-R  *SAPK2*-HA-F  *SAPK2*-HA-R  *NTRF1*-qF  *NTRF1*-qR  *SAPK2*-qF  *SAPK2*-qR  *OsGAmyb*-qF  *OsGAmyb*-qR  *OsRR24*-qF  *OsRR24*-qR  *OsC4*-qF  *OsC4*-qR  *OsPKS1*-qF  *OsPKS1*-qR  *CYP703A3*-qF  *CYP703A3*-qR  *OsABCG26*-qF  *OsABCG26*-qR | acaattacaggtaccgcggATGAATGCGGACAACTTCACGC  cgagctgcacgccgccCTCTTCGTCCATGCCGTCC  acaattacaggtaccgcggATGGAGAGGTACGAGGTGATCAA  cctcgcccttgctcaccatCAATGCGCACACGAAGT  acgggggacgagctcggtaccATGAATGCGGACAACTTCACGCACAAG  cgcgtacgagatctggtcgacCTCTTCGTCCATGCCGTCCTCG  tacgcgtcccggggcggtaccATGGAGAGGTACGAGGTGATCAA  acgaaagctctgcaggtcgacCAATGCGCACACGAAGT  gaacacgggggactctagaATGAATGCGGACAACTTCAC  tagtccatgtcgagtcgacaCTCTTCGTCCATGCCGTCC  acgacgtcccagactacgctATGGAGAGGTACGAGGTGAT  aagttcttctcctttactagtcagatctttaCAATGCGCACACGAAGTCGC  TGGATCGAGAAGAGGGTGGT  ACCTGGATGAGGATGTCCGA  GCAAACCCAGAGCAACGAATTACC  TCTCGATCGGCAGGTTCTTGAG  CAGACGCTACAGCAGATTCTTGGC  AACCGGCTTATCTCCATGCACTAC  GTTAACCAGCTTGGCATCGAGAAG  CCTGCTTGCATCAGTGCTCAAC  GCCAGATCAACTGCTCTGCCTAAG  AAGACAACGACGGCTTCGTCAAC  ACTTTGCAAGACCACCACAGTGAG  GTAGCACCAAGCTCAAGCACTG  GAGTGCATCCCTTGATGATG  ACTCGTTGGTCACCGATGAT  TGGCCTCTGAAGCTTGTCAGTTC  GAGGAAATGATCCGATGGATTGCG |

**Table S2. Primers used in this study**

**Table S2. (Continued) Primers used in this study**

| **Primer name** | **Primer sequence (5’-3’)** |
| --- | --- |
| *OsSAPK6*-qF | TGTCGCGAATCCTGCAAAGAG |
| *OsSAPK6*-qF | CCGCGGCAAGTTCTTCATAAACC |
| *OsPR4a*-qF | GGCAAGTGTATCCAGGTGAAGAAC |
| *OsPR4a*-qR | TTGCTGCATTGGTCAACAATCCTC |
| *OsbZIP23*-qF | GATGGAATTGGAAGCTGAGGTAGC |
| *OsbZIP23*-qR | TCGGCTCATTCTCTCTAGAACCTC |
| *OsPP2C49*-qF | TGTGATTGGTGGATGGTCAGAAC |
| *OsPP2C49*-qR | TCTAATCGCTGATCGCCTCAATCC |
| *OsbZIP72*-qF | CGCAAGCAGGCTTACACATTGG |
| *OsbZIP72*-qR | GCCTGTTTCCTCTCCAATTCCTTG |
| *OsMLP423*-qF  *OsMLP423*-qR  *OsPP45*-qF  *OsPP45*-qR  *OsNCED3*-qF  *OsNCED3*-qR  *OsUGT3*-qF  *OsUGT3*-qR  *OsRBOH9*-qF  *OsRBOH9*-qR  *OsRBOH8*-qF  *OsRBOH8*-qR  *OsRac1*-qF  *OsRac1*-qR  *OsRBOH6*-qF  *OsRBOH6*-qR  *OsFAH1*-qF  *OsFAH1*-qR  *OsMT2b*-qF  *OsMT2b*-qR  *OsLG3*-qF  *OsLG3*-qR  *OsFAH2*-qF  *OsFAH2*-qR  *OsRACK1A*-qF  *OsRACK1A*-qR  *OsCDPK5*-qF  *OsCDPK5*-qR | TTCGCATTCATCAGCTGGTAGG  ACCTTGGACGCCATGATCTCTC  TGCAGCAATGCTACCTCATCC  ATACAGCCCTGCATTGATTCCTTC  TCAGCTGAGTCCACACTAGTCC  TGGGCCCTCTCTCTCTCTATCTTG  TCAGCTATCTCGGATACCGTCAC  CAGCTGATCGACATCTTTGAGTGG  CATGGAGCACCATTCAAGGTTCC  TCCTTGACGATGCTGATGAGAGG  GTCTCCAAACCTTCTTCGACATGG  CTCCTCTTCTGTGATCCTGCCATC  GCAGCTCCAAGACACAGAGAAAC  TGGAGGTTGAAGCACCACTTTG  GCGTTCCGAGAAGAGCTTCATTTG  TCTACAAGCAACCTTGGTGGACTC  GCAGCTATCTTGTGCTTTCCGTTC  GGCTGCCCATGATGCAGATAGTAG  TGGATGCAGATGCAAGATGTTCCC  ATTCCTCCAGAGCTCGCCTTGTTG  TCGAGCAGTTCGACGATGACTC  GACACGAGATTTGGACGCGAAC  GGACTCACTCAGGCTTGTATTCCC  AACGCTACCAGCTTCCAGAACG  TCCCTGTCTCCAAGAACCAGATG  TGTAACCAGCATAGAGGGTGCTTC  TGTCTGTGTTCTTCAAGCCTGGTC  GCTTCTGGTCCGTAGCATTTACGC |
